# Aspiration versus stent retriever for posterior circulation stroke: A meta‐analysis

**DOI:** 10.1111/cns.14045

**Published:** 2022-12-13

**Authors:** Xiumei Guo, Yu Xiong, Xinyue Huang, Zhigang Pan, Xiaodong Kang, Chunhui Chen, Jianfeng Zhou, Cui'e Wang, Shu Lin, Weipeng Hu, Lingxing Wang, Feng Zheng

**Affiliations:** ^1^ Department of Neurology The Second Affiliated Hospital, Fujian Medical University Quanzhou China; ^2^ Department of Neurosurgery The Second Affiliated Hospital, Fujian Medical University Quanzhou China; ^3^ Centre of Neurological and Metabolic Research The Second Affiliated Hospital of Fujian Medical University Quanzhou China; ^4^ Diabetes and Metabolism Division Garvan Institute of Medical Research Sydney New South Wales Australia

**Keywords:** aspiration, posterior circulation stroke, stent retriever, thrombectomy

## Abstract

**Aims:**

New thrombectomy strategies have emerged recently. Differences between posterior circulation stroke management via aspiration and stent retriever remain to be evaluated. We compared the safety and efficacy of aspiration and stent retriever in treating posterior circulation stroke.

**Methods:**

Three databases (PubMed, Embase, and Cochrane Library) were systematically searched for studies comparing aspiration and stent retriever in patients with posterior circulation stroke. The modified Newcastle‐Ottawa scale was used to assess the risk of bias. A random‐effects model was used.

**Results:**

Fifteen cohort studies with 1451 patients were included. Pooled results showed a significant difference in total complication (odds ratio [OR] 0.48, 95% confidence interval [CI] [0.30, 0.76], *p* = 0.002). successful recanalization (1.85, [1.30, 2.64], *p* = 0.0006), favorable outcome (1.30, [1.02, 1.67], *p* = 0.04), procedure duration (−22.10, [−43.32, −0.88], *p* = 0.04), complete recanalization (4.96, [1.06, 23.16], *p* = 0.009), and first‐pass effect (2.59, [1.55, 4.32], *p* = 0.0003) between the aspiration and stent retriever groups, and in favor of aspiration. There was no significant difference in the outcomes of rescue therapy (1.42, [0.66, 3.05], *p* = 0.37) between the two groups.

**Conclusion:**

Patients with posterior circulation stroke receiving treatment with aspiration achieved better recanalization, first‐pass effect, and shorter procedure time. Aspiration may be more secure than a stent retriever.

## Graphical abstract

Patients with posterior circulation stroke receiving treatment with aspiration than that with a stent retriever achieved better recanalization, first‐pass effect, and shorter procedure time. Aspiration may be more secure than a stent retriever.

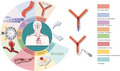

## 1 INTRODUCTION

In the past few years, the safety and efficacy of endovascular treatment in patients with ischemic stroke based on large vessel occlusion (LVO) in anterior circulation stroke has been proven in many randomized controlled trials, predominantly with the stent retriever as a treatment device.^1^, ^2^, ^3^, ^4^ However, randomized trials have failed to show efficacy in the posterior circulation.^5^, ^6^ In addition, at the initiation of the Multicenter Randomized Clinical Trial of Endovascular Treatment for Acute Ischemic Stroke in the Netherlands registry,^7^ stent retrievers were the most widely used devices in clinical practice. Over time, aspiration catheters have been increasingly used. In 2018, the numbers of patients treated with stent retriever and aspiration were almost equal.^7^ Since aspiration can safely and quickly recanalize with a few material resources, this technique has increasingly become the first‐line strategy in treating ischemic stroke.^8^ Findings from relevant randomized controlled trials have suggested that direct aspiration as the first‐line technique conferred functional outcomes comparable with those of stent retriever thrombectomy. However, patients with an ischemic stroke of the posterior circulation were omitted.^9^, ^10^ Compared with the anterior circulation, posterior circulation lacks collateral and is, therefore, more susceptible to prolonged time and reperfusion. Previous studies have reported higher rates of successful recanalization and shorter procedural duration in patients with posterior circulation stroke treated with aspiration; however, they could not adequately demonstrate the safety between aspiration and stent retriever.^11^, ^12^

The thrombectomy technique has been rapidly developed with new devices and different strategies in recent years to improve the reperfusion success rate and reduce distal embolism. Promising techniques using large‐bore aspiration catheters and/or new‐generation stent retrievers have shown promising results, with high reperfusion rates.^13^ However, it remains unclear whether aspiration as a first‐line technique is superior to a stent retriever regarding recanalization rate and clinical outcome. In this meta‐analysis, we aimed to compare the safety and efficacy of direct aspiration with that of a stent retriever in treating patients with posterior circulation stroke and optimize the interventional strategy for mechanical thrombectomy in posterior circulation stroke.

## 2 METHODS

### 2.1 Search strategy

This meta‐analysis was performed according to the recommendations and guidelines of the Preferred Reporting Items of Systematic Reviews and Meta‐Analyses (PRISMA).^14^ Two authors independently performed a comprehensive literature search. Studies were collected from PubMed, Embase, and Cochrane databases from inception to April 2022 with no language restriction, using the following terms: “posterior circulation,” “basilar,” “stroke,” “thrombectomy,” “aspiration,” and “stent retriever.” (The complete search strategy is provided in the Appendix S1). In addition, the list of references for each study included in the analysis was manually searched to identify other publications. All included studies met the inclusion criteria. Any discrepancies discovered during the literature search were resolved by consulting with the corresponding author.

### 2.2 Outcomes

The primary outcomes of this meta‐analysis included reperfusion metrics and complications. Reperfusion metrics included (a) successful recanalization (assessed using the degree of revascularization after endovascular procedure, which was defined using a modified thrombolysis in cerebral infarction (mTICI) score of 2b or 3, corresponding to reperfusion of approximately 50% of the affected vascular territory)^15^; (b) complete recanalization (mTICI = 3); and (c) first‐pass effect (defined as first‐pass maneuver achieving mTICI = 3). The outcomes of complications, including mortality (evaluated at 90 days); hemorrhagic complications, including symptomatic intracranial hemorrhage (SICH, defined as any intracranial hemorrhage associated with a worsening of the National Institutes Health Stroke Scale (NIHSS) score by ≥4 within 24 h, according to the European Cooperative Acute Stroke Study‐II definition),^16^ parenchymal hematoma, subarachnoid hemorrhage, embolization to new territory, and vessel perforation.

Secondary outcomes included (a) favorable outcome (assessed using the modified Rankin Score [mRS]) at 3 months after stroke onset. The mRS is one of the most widely used endpoints for stroke severity in clinical acute stroke trials and is valid and reliable.^17^ It captures the patient's functional outcome on an ordinal scale ranging from 0 to 6, where zero indicates no symptoms and 6 denotes a patient's death.^18^ We defined favorable outcomes as an mRS score of 0–2 at 3 months; (b) procedure time; and (c) rescue therapy (defined as the use of another endovascular strategy after failure of the first‐line treatment).

### 2.3 Study selection

Two researchers independently screened all retrieved literature. The criteria for selecting the studies were as follows: (a) randomized controlled trials or observational cohort studies; (b) comparison of the safety and efficacy of aspiration and stent retriever in treating posterior circulation stroke; and (c) studies reporting at least one outcome. Exclusion criteria were: (1) studies that used non‐human subjects; (2) the data in the study could not be extracted; (3) studies that were not comparative studies, such as case reports, reviews, meetings, letters, surveys, or satisfaction studies; (4) studies only equipped with a single‐arm design; and (5) studies not meeting inclusion criteria.

### 2.4 Data extraction

Two authors independently extracted baseline characteristics, primary outcome, and secondary outcomes from the included studies. The baseline data and primary and secondary outcomes were extracted from each eligible study. Baseline data included first author, year of publication, sample size, study design (prospective or retrospective study), sex, NIHSS score on admission, time from onset to groin puncture, distribution of occlusion sites, aspiration, and stent retriever devices. Since stroke etiology, pre‐imaging selection and selection criteria for thrombectomy may greatly affect the outcomes of thrombectomy,^19^, ^20^, ^21^ the relevant data of these factors has also been collected. Any question was communicated and resolved by the corresponding author.

### 2.5 Critical appraisal

Two authors independently completed the quality assessment of the included studies. The Newcastle–Ottawa Scale (NOS) was used to assess the quality of observational cohort studies included in this meta‐analysis, which gives a score out of a possible total of nine stars.^22^ The Scale rates were studied under the selection, comparability, and outcome categories. Four stars were available in the selection category, two in the comparability category, and three possible stars could be achieved in the outcome category. The maximum number of stars was nine, and studies were graded as “high quality,” with scores of 6+, “moderate” with scores of 4–5, and “weak” with scores of 0–3. Furthermore, a Newcastle–Ottawa Scale score of >7 indicated high quality.

### 2.6 Statistical analysis

The quality of the included studies was carefully evaluated. Differences in opinions were resolved through discussion. Statistical heterogeneity was measured using the c^2^ and I^2^ statistics. According to the recommendation of the Cochrane Statistical Methods Group,^23^ a significance level of heterogeneity was set at a *p*‐value of 0.1, and the I^2^ statistic was interpreted as follows: 0%–40%, low heterogeneity; 30%–60%, moderate heterogeneity; 50%–90%, substantial heterogeneity; and 75%–100%, considerable heterogeneity. Statistically significant heterogeneity was present at *p* < 0.1 and I^2^ > 50%. In these cases, a sensitivity analysis was employed to identify the robustness of the results. In addition, we used a random‐effects model to pool the results of the primary studies to judge their appropriateness. Data management and analysis were performed using Cochrane Collaboration's Review Manager (RevMan 5.4).

## 3 RESULTS

### 3.1 Study inclusion

**FIGURE 1:**
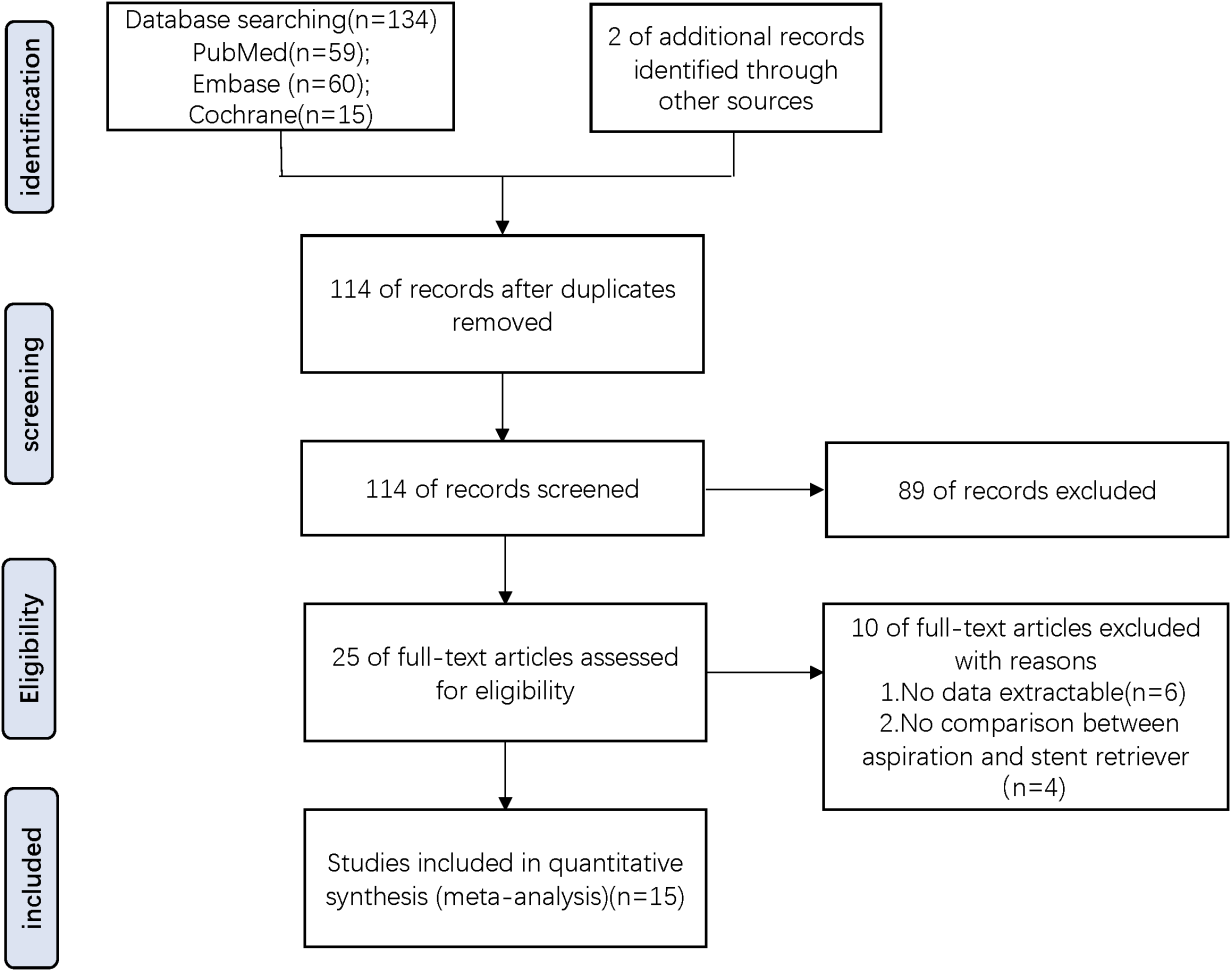
Flow chart showing search strategy.

A total of 136 potentially eligible articles were identified. Among these, 114 were obtained after removing duplicates. Subsequently, 89 non‐pertinent articles were excluded by screening titles and abstracts. Of the remaining 25 articles, full texts were accessed, and 15 articles comparing the clinical outcomes of aspiration and stent retriever for patients with posterior circulation stroke were included. Of the 25 articles, 10 were excluded because of either no extractable data (*n* = 6) or because no comparison was made between aspiration and stent retrievers (*n* = 4; Figure 1).

Until the literature search, no randomized controlled trials have been published on this topic. All 15 studies^12^, ^24^, ^25^, ^26^, ^27^, ^28^, ^29^, ^30^, ^31^, ^32^, ^33^, ^34^, ^35^, ^36^, ^37^ with 1451 patients were cohort studies (six prospective and nine retrospective studies). These studies were published between 2014 and 2022. A total of 549 and 852 patients were treated with first‐line aspiration and a stent retriever, respectively. The characteristics and detailed quality assessments of the 15 included studies are shown in Table 1

**TABLE 1:** Baseline characteristics of the included studies

Author and publication year	Study design and period	Total (*n*)	Patients (*n*)		Devices		Age (year)		Sex (Male)		Time from onset to groin puncture (min)		Initial NIHSS		Location (%)		NOS score
			ASP	SR	ASP	SR	ASP	SR	ASP	SR	ASP	SR	ASP	SR			
Bernsen 2022^35^	Prospective 2014–2018	205	71	134	NA	NA	60 (51–71)	66 (58–79)	41 (58%)	74 (55%)	230 (155–367)	262 (128–383)	19 (12–32).	20 (10–29)	NA		8
Onodera 2021^25^	Retrospective 2014–2016	34	23	11	NA	NA	74.78 ± 11.5	73.09 ± 13.1	17 (73.9%)	7 (63.6%)	NA	NA	21.7 ± 9.65.	21 ± 9.75	BA	88.2%	5
															VA	11.8%	
Abdelrady 2022^37^	Retrospective 2015–2019	128	33	35	NA	NA	67.5 ± 11	65.97 ± 13.7	19 (58)	20 (57)	314 (203–390)	250 (150–400)	14 (8–19.5)	18 (12–40)	VA	11%	5
															BA	89%	
Baik 2021^36^	Prospective 2013–2019	161	43	118	NA	NA	NA	NA	NA	NA	NA	NA	NA	NA	NA		7
Choi 2020^34^	Retrospective 2016–2019	50	34	16	5MAX ACE. 68 catheter	NA	65.25 (51–84)	69.38 (18–89)	10 (62.5)^a^		125 (60–330)	140 (75–375)	19.5 (13.5–22.25).	21.5 (13.25–26.75)	NA	5	
Kaneko 2021^30^	Retrospective 2015–2019	73	38	21	NA	NA	77 (68–84)^a^		47 (64.4)^a^		NA	NA	24 (13–30)^a^		V‐BA	5.5%	5
															BA	94.5%	
De La Riva 2018^33^	Prospective 2015–2016	61	21	40	NA	NA	NA	NA	NA	NA	NA	NA	5.4 (3.2–9.3)	4.8 (3.5–6.8)	BA	80.3%	6
															VA	0.05%	
															PCA	0.15%	
Giorgianni 2018^32^	Retrospective 2010–2015	87	27	60	NA	NA	68 (57–76)^a^		65 (63.7)^a^		NA	NA	17^a^		BA	100%	6
Gory 2018^12^	Prospective 2010–2016	100	46	54	5MAX ACE. 5MAX/Other	Solitaire Trevo/other	61 (53–71)	67 (53–78)	27 (58.7)	34 (63)	342 (241–440).	276 (98–355)	6 (6–9).	8 (6–8)	NA	6	
Haussen 2018^31^	Prospective 2010–2017	87	20	48	Penumbra 5MAX /064/068	Solitaire Trevo	NA	NA	NA	NA	NA	NA	NA	NA	NA	4	
Kang 2018^29^	Prospective 2010–2016	212	67	145	NA	Solitaire	71 (64–78)^a^		71 (64–78)^a^		295 (224–424)^a^		17 (10–24)^a^		BA	100%	9
Lee 2018^28^	Retrospective 2012–2016	15	8	7	Penumbra5Fr Catheter	Solitaire/Trev	NA	NA	NA	NA	NA	NA	NA	NA	NA	4	
Li 2017^27^	Retrospective 2012–2016	68	40	71	5MAXACE	Solitaire AB/FR	NA	NA	NA	NA	NA	NA	NA	NA	NA	6	
Mokin 2016^26^	Retrospective 2012–2015	100	42	58	NA	Solitaire FR Trevo	63.5 ± 14.2^a^		67 (67)^a^		562 ± 466^a^		19.2 ± 8.2^a^		NA	7	
Son 2014^24^	Retrospective 2011–2013	31	18	13	054Reperf/catheter	Solitaire AB	66.4 ± 11.4	69.8 ± 10.4	14 (77.8)	7 (53.8)	109.2 ± 65.4	117.4 ± 57.0	21.3 ± 9.7	27.3 ± 11.0	NA	5	

Author and publication year	Country	Pre‐treatment imaging modality	Stroke etiology		Selection criteria for thrombectomy
			ASP	SR	
Bernsen 2022^35^	Netherlands	CTA	LAA 34%	LAA 29%	Patients were treated according to national guidelines for the treatment of acute ischemic stroke, including intravenous thrombolysis, if indicated.
			CE 20%	CE 31%	
			AD 10%	AD 8%	
			Other 37%	Other 32%	
Onodera 2021^25^	Japan	MRA	NA		The decision to initiate MT in the endovascular approach in any given patient had been left to attending neurointerventional specialist's discretion in agreement with the stroke care team and the patient's family.
Abdelrady 2022^37^	France	MRA and DSA	Emboli 79%	Emboli 59%	The choice of revascularization strategy was left to the discretion of the neurointerventionist in conformity with local institutional interdisciplinary stroke guidelines
			ICAS 9%	ICAS 23%	
			Tandem 12%	Tandem18%	
Baik 2021^36^	Korea	CTA	NA	NA	NA
Choi 2020^34^	Korea	CT, CTA, or MRI	NA	NA	The decision to initiate endovascular treatment in any given patient with BAO on presentation was left to the attending neurointerventional specialist's discretion in agreement with the neurologist according to neurological guidelines.
Kaneko 2021^30^	Japan	CCA	NA	NA	Each center could decide the strategy for revascularization and select the devices deemed most appropriate for treatment.
De La Riva 2018^33^	Spain	NA	NA	NA	NA
Giorgianni 2018^32^	Italy	CTA, MRA	LAA 32.6%	LAA 33.3%	BAO was confirmed by angiography and all patients were subject to endovascular treatment. Patients affected by BAO that had been treated with rt‐PA only and those who did not receive any acute treatment were excluded from the study.
			CE 37.2%	CE 37.2%	
			Other 30.2%	Other30.2%	
Gory 2018^12^	France	MRI, CT, CTA	NA	NA	The decision to treat or not to treat any given patient with BAO on presentation was left to the attending neuroendovascular specialist's discretion in agreement with the stroke team caring for the patient and the patient's family.
Haussen 2018^31^	Korea	NA	NA	NA	NA
Kang 2018^29^	Korea	NA	CE 47.6%^a^		At all centers, patients were considered eligible for thrombectomy if the thrombectomy could be started within 12 h of the estimated time of stroke onset and had an angiographically confirmed occlusion in the basilar artery. Patients were excluded from thrombectomy when pretreatment imaging showed extensive ischemic changes in the brain stem or the stroke was considered mild, based on an admission NIHSS score of 3 or less.
			LAA 38.7%		
			Other 13.7%		
Lee 2018^28^	Korea	CTA, MRA, CCA	NA	NA	MT was considered in patients with a NIHSS score of 4 or greater, older than 18 years, with definite occlusion of the basilar artery or PCA who arrived at the hospital within 24 h from onset.
Li 2017^27^	Germany	CA (DSA)	NA	NA	Authors typically selected patients for endovascular treatment of these patients based on the Pc‐ASPECTS cutoff value of 6. Patients with Pc‐ASPECTS ≤ 6 was also treated with endovascular therapy on an individual case by case basis after discreet consideration about patient conditions, such as patient age, stroke severity, prior mRS, collateral status, and so on.
Mokin 2016^26^	American	CT, CTA, MRI	NA	NA	NA
Son 2014^24^	Korea	CTA, MRA	LAA 38.9%	LAA 30.8%	NA
			CE 55.6%	CE 69.2%	
			Other5.6%		

*Note*: Newcastle Ottawa scale (NOS), which gives a score out of a possible total of 9 stars. The scale rates studies under the categories of selection, comparability, and outcome. Four stars are available from the selection category, 2 stars are available from the comparability section, and a possible 3 stars can be achieved in the outcome categories.

Abbreviations: ASP, aspiration; BA, basilar artery; BAO, basilar artery treatment; CCA, conventional contrast angiography; CE, Cardio embolism; CT, computed tomography; CTA, computed tomography angiography; DSA, digital subtraction; LAA, Large artery atherosclerosis; MRA, magnetic resonance angiography; MRI, magnetic resonance imaging; MT, mechanical thrombectomy; NA, not available; NIHSS, national institutes of stroke scale; PCA, posterior cerebral artery; SR, stent retriever; VA, vertebral artery.

^a^ No separate value.

### 3.2 Primary outcome

#### 3.2.1 Successful recanalization

**FIGURE 2:**
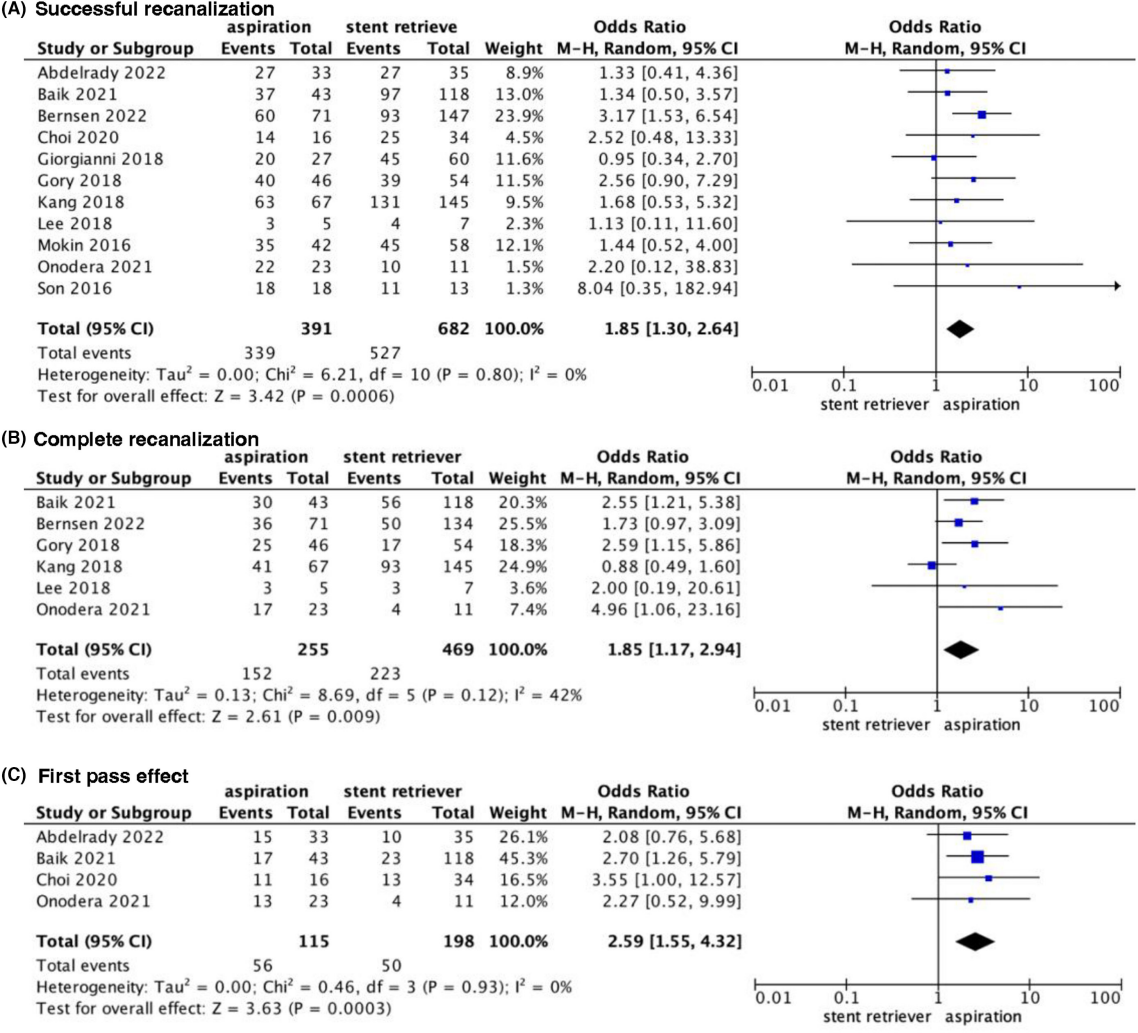
Forest plot for Forest plot for successful recanalization, complete recanalization, and first‐pass effect.

Eleven studies^12^, ^24^, ^25^, ^26^, ^28^, ^29^, ^32^, ^34^, ^35^, ^36^, ^37^ reported successful recanalization. The pooled analysis showed a significant difference between the two groups (OR, 1.85; 95% CI [1.30, 2.64], *p* = 0.0006; Figure 2A) in favor of aspiration. Further subgroup analysis in patients with basilar artery occlusion showed a greater tendency for successful recanalization among patients receiving aspiration compared with those receiving stent retriever (OR 1.56, 95% CI [1.00, 2.45], *p* = 0.05; Figure S1).

#### 3.2.2 Complete recanalization

Six studies^12^, ^25^, ^28^, ^29^, ^35^, ^36^ reported complete recanalization. The pooled results showed a significant difference between aspiration and stent retriever (OR 1.85, 95% CI [1.17–2.94], *p* = 0.009; Figure 2B) in favor of aspiration. Subgroup analysis of patients with basilar artery occlusion showed a significant difference favoring aspiration (OR 2.11, 95% CI [1.03, 4.33], *p* = 0.04; Figure S1B). A sensitivity analysis was used to assess findings' robustness due to the existence of significant heterogeneity (*p* = 0.03; I^2^ = 62%). After the exclusion of one study,^29^ heterogeneity significantly decreased (*p* = 0.54; I^2^ = 0%), with the significance in favor of aspiration (OR 2.82, 95% CI [1.69, 4.69], *p* < 0.0001).

#### 3.2.3 First‐pass effect

Four studies^25^, ^34^, ^36^, ^37^ reported the first‐pass effect. The pooled results showed that compared with a stent retriever, patients treated with aspiration had higher rates of first‐pass effect (OR 2.59, 95% CI [1.55, 4.32], *p* = 0.0003; Figure 2C). Based on the three studies,^34^, ^36^, ^37^ subgroup analysis showed a significant difference in the first‐pass effect in patients with basilar artery occlusion in favor of aspiration (OR 2.63, 95% CI [1.52, 4.55], *p* = 0.0005; Figure S1C).

#### 3.2.4 Complications

**FIGURE 3:**
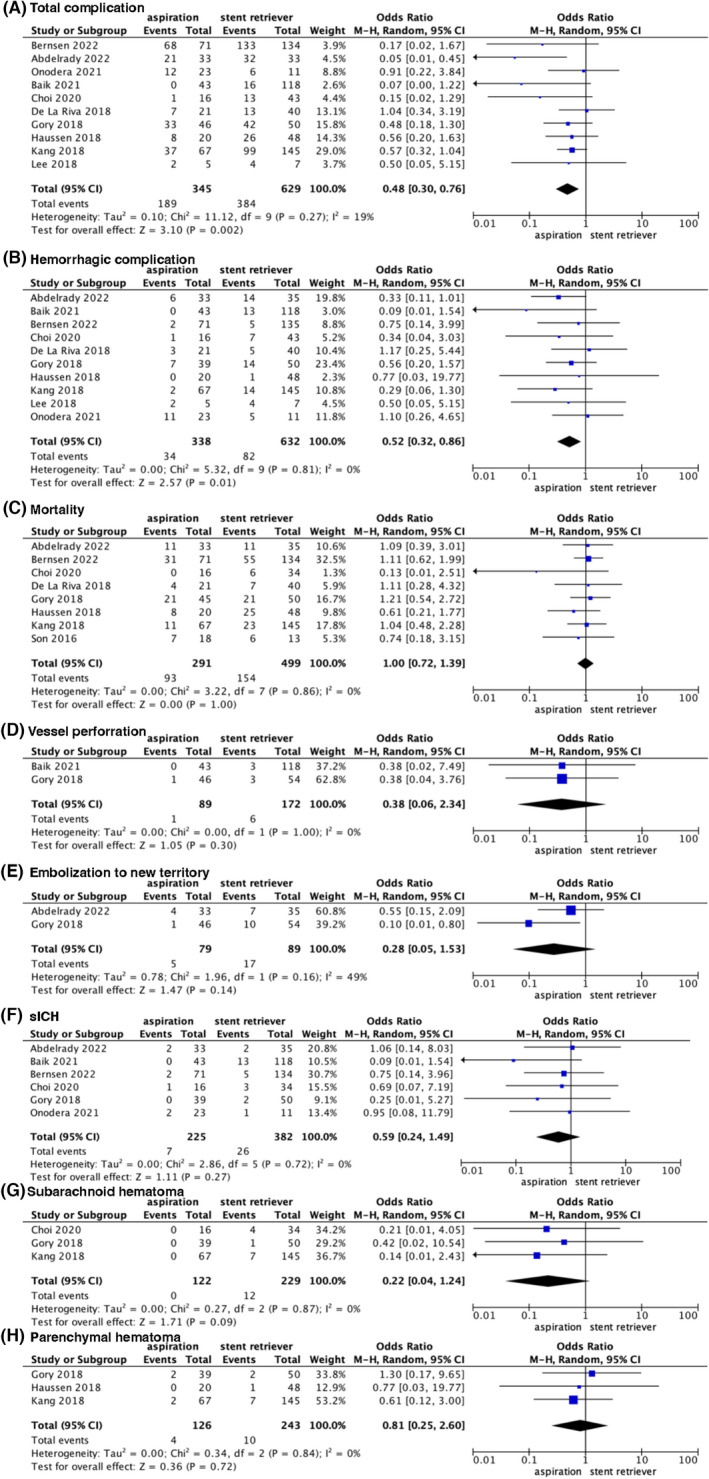
Forest plot for total complication, subgroup for various complications, and subgroup for different hemorrhagic types. sICH, symptomatic intracranial hemorrhage.

Ten studies^25^, ^28^, ^29^, ^31^, ^33^, ^34^, ^35^, ^36^, ^37^ recorded complications, while six did not. The pooled analysis showed that compared with stent retriever, aspiration had lower rates of complications (OR, 0.48; 95% CI [0.30, 0.76], *p* = 0.002; Figure 3A). Ten studies reported hemorrhagic complications. The pooled data showed a statistically significant difference in the outcome of hemorrhagic complications between the two groups, in favor of the aspiration group (OR, 0.52; 95% CI [0.32, 0.86], *p* = 0.01; Figure 3B). No significant difference was detected in other complications, including mortality (OR 1.00, 95% CI [0.72, 1.39], *p* = 1.00; Figure 3C), vessel perforation (OR 0.38, 95% CI [0.06, 2.34], *p* = 0.30; Figure 3D), and embolization to new territory (OR 0.28, 95% CI [0.05, 1.53], *p* = 0.14; Figure 3E). For each hemorrhagic complication, there was no significant difference in SICH (OR 0.59, 95% CI [0.24, 1.49], *p* = 0.27; Figure 3F), subarachnoid hemorrhage (OR 0.22, 95% CI [0.04, 1.24], *p* = 0.09; Figure 3G), and parenchymal hematoma (OR 0.81, 95% CI [0.25, 2.60], *p* = 0.72; Figure 3H). As the data on complications regarding basilar artery occlusion were sufficiently provided, subgroup analysis was conducted. The outcome of total complications (OR 0.41, 95% CI [0.23, 0.71], *p* = 0.002; Figure S2A) and hemorrhagic complications (OR 0.40, 95% CI [0.22, 0.72], *p* = 0.002; Figure S2B) from subgroup analysis in patients with basilar artery occlusion showed significant differences in favor of aspiration. No significant difference was detected in other complications, including mortality (OR 0.94, 95% CI [0.61, 1.43] *p* = 0.76; Figure S2C), vessel perforation (OR 0.38, 95% CI [0.06, 2.34], *p* = 0.30; Figure S2D), and embolization to new territory (OR 0.28, 95% CI [0.05, 1.53], *p* = 0.14; Figure S2E). In patients with basilar artery occlusion, subgroup analysis showed no significant difference in each hemorrhagic type, including SICH (OR 0.47, 95% CI [0.14, 1.61], *p* = 0.23; Figure S2F), subarachnoid hemorrhage (OR 0.22, 95% CI [0.04, 1.24], *p* = 0.09; Figure S2G), and parenchymal hematoma (OR 0.81, 95% CI [0.25, 2.60], *p* = 0.72; Figure S2H).

### 3.3 Secondary outcomes

#### 3.3.1 Favorable outcome

**FIGURE 4:**
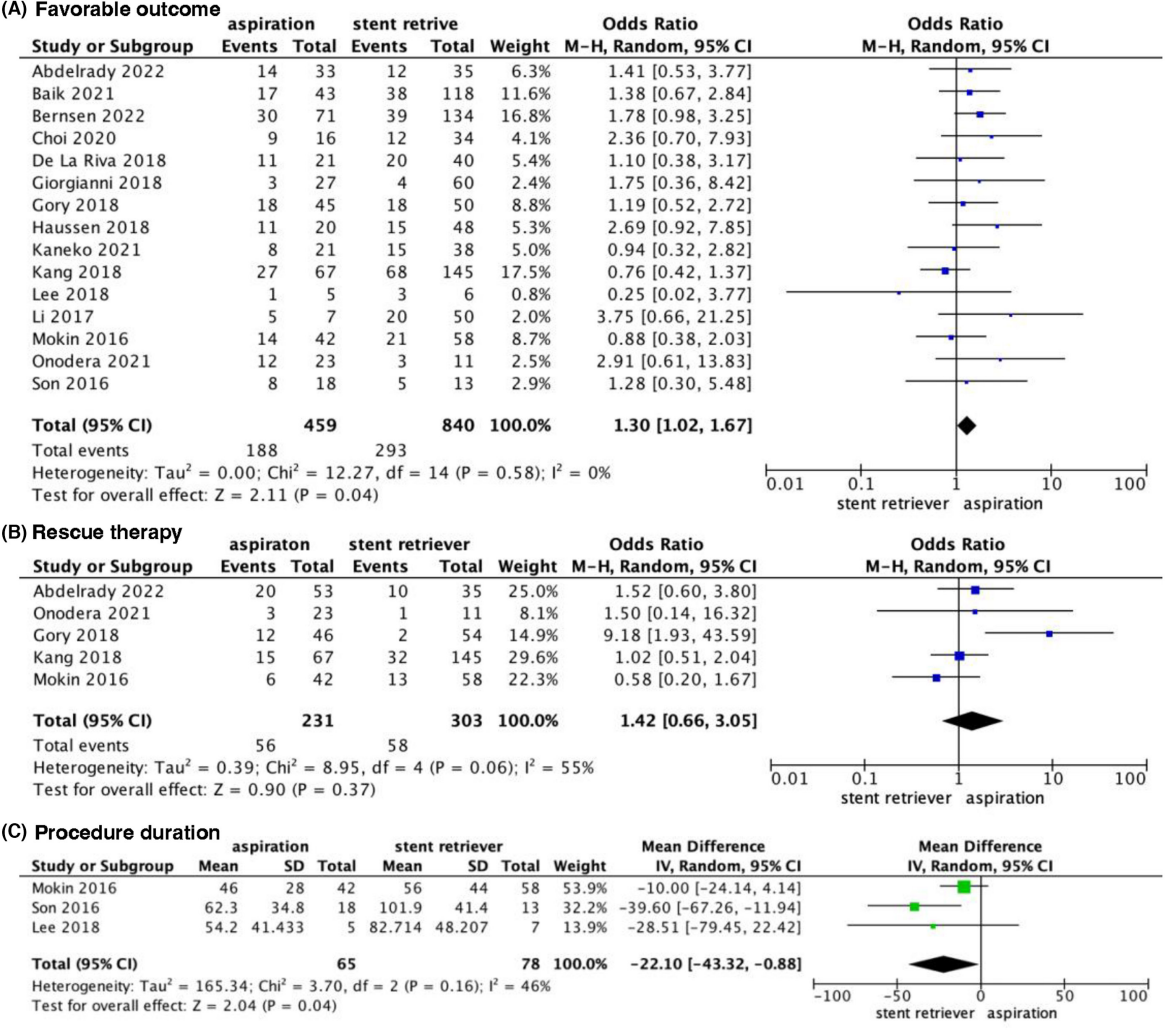
Forest plot for secondary outcomes.

Fifteen studies^12^, ^24^, ^25^, ^26^, ^27^, ^28^, ^29^, ^30^, ^31^, ^32^, ^33^, ^34^, ^35^, ^36^, ^37^ reported favorable outcomes. The pooled results showed a significant difference between the two groups (OR 1.30; 95% CI [1.67, 1.67], *p* = 0.04; Figure 4A) in favor of aspiration. A subgroup analysis of favorable outcomes in basilar artery occlusion was conducted using data from 11 studies.^12^, ^24^, ^27^, ^28^, ^29^, ^30^, ^31^, ^32^, ^34^, ^36^, ^37^ The pooled results did not show significant difference between the two groups (OR 1.24, 95% CI [0.92, 1.68], *p* = 0.16; Figure S3A).

#### 3.3.2 Rescue therapy

Five studies^12^, ^25^, ^26^, ^29^, ^37^ reported the proportion of rescue therapies. No significant difference was detected between the two techniques (OR, 1.42; 95% CI [0.66, 3.05], *p* = 1.42; Figure 4B). There was no significant difference detected in the subgroup analysis in patients with basilar artery occlusion between the two groups (OR, 2.08; 95% CI [0.73, 5.89], *p* = 0.17; Figure S3B). Owing to the substantial heterogeneity in the two outcomes above, (*p* = 0.06; I^2^ = 55%) and (*p* = 0.04; I^2^ = 70%), respectively, further sensitivity analyses were employed to assess the robustness of the findings. After exclusion of one trial^12^ for both outcomes, the heterogeneity significantly decreased (*p* = 0.59, I^2^ = 0% and *p* = 0.35, I^2^ = 0%, respectively), with no significant difference in the pooled results (OR 1.03, 95% CI [0.63, 1.66], *p* = 0.92 and OR 1.25, 95% CI [0.72, 2.17], *p* = 0.43, respectively).

#### 3.3.3 Procedure duration

Procedure duration^24^, ^26^, ^28^ was a continuous variable. The pooled results showed a significant difference between aspiration and stent retriever in favor of aspiration (OR −22.10, 95% CI [−43.32, −0.88], *p* = 0.04; Figure 4C).

### 3.4 Subgroup analysis based on East‐Asian studies and Non‐East Asian studies

The difference in the etiology of LVO might greatly affect the outcomes of thrombectomy in posterior circulation LVO.^21^ It is well known that underlying intracranial atherosclerotic disease (ICAD) is more common in posterior circulation LVO than anterior circulation LVO.^38^, ^39^, ^40^ In addition, ICAD has been identified as the cause of lesions in 25%–40% of patients with acute basilar artery occlusion in Chinese and Korean studies.^41^ Therefore, separate meta‐analyses were performed after separating studies into two categories, East‐Asian and non‐East Asian studies. The results of the subgroup analysis are provided in Table 2 and the forest plot in Figure S4.

**TABLE 2:** Outcomes based on East‐Asian group and Non‐East Asian group

Outcomes	No. of studies		No. of patients, *n* (%)		Pooled results (OR [95% CL])		*p* value		I^2^ (%)	
	E.A.	Non‐E.A.	E.A.	Non‐E.A	E.A.	Non‐E.A.	E.A.	Non‐E.A.	E.A.	Non‐E.A.
Successful recanalization	5	6	466 (28.9)	604 (71.1)	1.68 [0.88, 3.18]	1.43 [0.95, 2.16]	0.11	0.08	0	0
Complete recanalization	3	3	385 (43.7)	473 (56.3)	1.50 [0.64, 3.52]	1.65 [1.03, 2.65]	0.35	0.04	59	28
First‐pass effect	2	3	211 (35.0)	307 (65.0)	2.91 [1.51, 5.58]	1.82 [1.13, 2.94]	0.001	0.01	0	0
Total complication	5	5	512 (54.0)	462 (46.0)	0.50 [0.31, 0.81]	0.45 [0.18, 1.11]	0.005	0.08	0	47
Hemorrhagic complications	5	5	512 (25.8)	458 (74.2)	0.31 [0.12, 0.83]	0.62 [0.35, 1.11]	0.02	0.11	0	0
Mortality	4	4	361 (34.2)	429 (65.8)	0.79 [0.45, 1.39]	1.13 [0.75, 1.70]	0.41	0.55	0	0
Vessel perforation	1	1	161 (37.2)	100 (62.8)	0.38 [0.02, 7.49]	0.38 [0.06, 2.34]	0.52	0.41	NA	0
sICH	2	4	211 (26.0)	390 (74.0)	0.29 [0.04, 2.36]	0.75 [0.26, 2.20]	0.25	0.60	25	0
Subarachnoid hematoma	2	1	280 (66.2)	89 (33.8)	0.64 [0.15, 2.67]	1.30 [0.17, 9.65]	0.54	0.80	0	NA
Parenchmal hematoma	2	1	262 (66.2)	89 (33.8)	1.30 [0.17, 9.65]	0.17 [0.02, 1.32]	0.09	0.60	0	NA
Favorable outcome	6	9	533 (42.2)	766 (57.8)	1.28 [0.78, 2.09]	1.39 [1.01, 1.92]	0.33	0.04	29	0
Rescue therapy	1	4	212 (29.2)	326 (70.8)	1.02 [0.51, 2.04]	1.80 [0.58, 5.55]	0.96	0.31	NA	65
Procedure duration	2	1	43 (45.7)	100 (54.3)	−36.84 [−61.15, −12.54]	−10.00 [−24.14, 4.14]	0.003	0.17	0	NA

Abbreviations: E.A., East‐Asia; Non‐E.A., Non‐East Asia; sICH, symptomatic intracranial hemorrhage; NA: not applicable.

## 4 DISCUSSION

Higher rates of successful recanalization and a shorter procedure duration were strongly associated with increased favorable outcomes.^42^, ^43^, ^44^ This meta‐analysis showed that aspiration might be more effective and safer for posterior circulation stroke than stent retriever, with a higher rate of successful recanalization, faster procedure duration, and higher probability of favorable outcomes. The subgroup analysis of basilar artery occlusion showed no significant difference in the outcomes of successful recanalization, which is different from that of a previous study.^45^ This may be due to the study by Giorgianni included in this meta‐analysis,^32^ which started in 2010 when most of the procedures were performed with old devices, and the recanalization rate was lower in the aspiration group than in the stent retriever group. As a result, the advantage of aspiration on the successful recanalization rate was weakened in this meta‐analysis. The pooled results showed that the procedure duration was significantly shorter in the aspiration group than in the stent retriever group. Compared with a stent retriever, aspiration is technically easier, and the aspiration catheter needs to be placed proximal to the thrombus without the micro guidewire and catheter passing the clot, as is the case with a stent retriever, which might optimize the final step by streamlining the process through rapid reperfusion, leading to better outcomes.^46^

For the new‐generation devices, a novel outcome measure was described, the first‐pass effect, which has emerged as a core metric of effective mechanical thrombectomy for acute ischemic stroke.^47^ In this meta‐analysis, statistical significance was reached in the outcome of the first‐pass effect between aspiration and stent retriever in patients with posterior circulation stroke, in favor of aspiration. Further subgroup analysis of basilar artery occlusion showed that aspiration was superior to stent retriever in this outcome. In addition, the North American Solitaire Acute Stroke (NASA) registry database^48^ reported that the first‐pass effect had a better impact on favorable outcomes and fewer complications in patients compared with final reperfusion of mTICI 3. Among the 15 studies in our meta‐analysis, when the initial thrombectomy technique failed, the interventionalists could switch to another method to achieve successful reperfusion. However, multiple manipulations may damage the blood vessel walls, contributing to higher complication rates.^48^ Therefore, patients in the aspiration group (achieved higher first‐pass effect rate) had better favorable outcomes, procedure duration, and lower complication rates than of those treated with a stent retriever.

Our findings also confirmed that treatment with aspiration was a more secure method than the stent retriever. Among the 10 studies^25^, ^28^, ^29^, ^31^, ^33^, ^34^, ^35^, ^36^, ^37^ that reported complications, nine studies favored aspiration therapy (except De La Riva et al.^33^ that favored stent retriever). However, except for the study by Abdelrady et al.^37^ none of the other eight studies showed a statistically significant difference in complications between the two devices. When pooled together, the pooled results showed that patients treated with aspiration had lower rates of complications than those treated with a stent retriever. However, our pooled results indicated fewer hemorrhagic complications in the aspiration group. It is worth noting that although there was no statistically significant difference in the complications of subarachnoid hemorrhage between the aspiration and stent retriever groups, there was a trend in favor of aspiration, suggesting that blind wire passage may lead to vascular perforation. This possibility is more dangerous in the basilar artery and perforator vessels of the proximal posterior circulation artery than in the anterior circulation, which causes more hemorrhagic complications.^34^ No significant difference was detected in other complications, including SICH, parenchymal hematoma, mortality, vessel perforation, and embolization to new territory.

The clinical outcome for patients with posterior circulation stroke are strongly associated with the underlying mechanisms of stroke.^21^ In the present analysis, only five studies reported the proportion of causative mechanisms (Table 1). Kang et al. compared the procedural characteristics and outcomes between first‐line stent retriever and aspiration in patients with emergent LVO due to underlying ICAD; they concluded that stent retrievers may be more suitable than aspiration in such patients.^49^ Furthermore, the included study by Abdelrady et al.^37^ showed that, due to the inability of aspiration to reduce the burden or fully retrieve the clot, patients with underlying ICAD with aspiration were more likely to switch to another rescue therapy. Moreover, there are few studies that report the efficacy and safety in posterior circulation stroke based on ICAD.^50^ In addition, no separate data based on different causative mechanisms was provided in the 15 studies included. Nevertheless, based on the etiological differences in ICAD, a further subgroup analysis was performed between East‐Asian and non‐East‐Asian studies. In our meta‐analysis, aspiration was still superior to stent retrievers in that it achieved complete recanalization and favorable outcomes in the non‐East‐Asian group. However, no significant difference was observed between aspiration and stent retrievers in terms of complete recanalization and favorable outcomes in the East‐Asian group, which were inconsistent with the primary pooled results. This may be explained by the contact problem in the aspiration system. Due to pre‐existing atherosclerosis within the vessel wall, most intracranial atherosclerotic stenosis segments are conical and irregular in shape; thus, it may be difficult for the tip of large‐bore aspiration catheter to make contact with the surface of a thrombus.^41^ Therefore, the advantage of aspiration in complete recanalization and favorable outcomes was reduced in East‐Asian groups. In contrast, pooled results of total complications, hemorrhagic complications, and procedure duration showed that aspiration was still superior in the East‐Asian group, consistent with the primary outcomes.

Various factors, including the NIHSS baseline score, mechanical thrombectomy devices, vascular occlusion site, and stroke pathogenesis, may affect the efficacy of mechanical thrombectomy in patients with posterior circulation stroke, which is also the reason for some heterogeneity among the included studies. In recent years, a new generation of mechanical thrombectomy techniques has been described, with good reperfusion results in the anterior circulation.^51^, ^52^, ^53^ ADAPT, a direct aspiration first‐pass technique in the posterior circulation, has become increasingly popular and is equipped with an innovative large‐bore aspiration catheter with better accessibility to the intracranial arteries and stronger suction forces to retrieve the thrombus. Patients treated with the ADAPT technique achieved a higher successful recanalization rate and more favorable outcomes at 90 days, demonstrating the superiority of ADAPT as a first‐line strategy for mechanical thrombectomy in the posterior circulation over the stent retriever.^25^, ^33^ Next‐generation aspiration catheters hold the potential to provide even better clinical outcomes.

In recent years, thrombectomy techniques have developed rapidly, including stent retriever assisted vacuum‐locked extraction (SAVE),^51^ proximal balloon occlusion, and direct thrombus aspiration during stent retriever thrombectomy (PROTECT),^54^ and Solumbra,^55^ which combines stent retriever thrombectomy with or without the use of an aspiration catheter. Among the combined approaches, a new method that combines PROTECT and SAVE was used in the study by Carlos et al.^56^ known as PROTECT‐PLUS, incorporating large‐bore aspiration catheters, associated with higher reperfusion rates, shorter procedure times, and lower need for rescue therapy with reduced complication rates. This represents a potential direction for future research.

Several crucial issues have been found from a thrombectomy for anterior circulation stroke, which are highly likely to be relevant to posterior circulation stroke: (1) tissue no‐reflow phenomenon despite complete recanalization, which is infrequent and may not substantially contribute to futile recanalization or stand as a critical therapeutic target^57^; (2) the relevance of collateral status in the prognosis of thrombectomy, which not only predicted the infarct volumes soon after stroke onset^58^ but also were strongly associated with less cerebral edema formation and favorable outcomes at 90 days^59^; and (3) the impact of age on the increased brain edema after thrombectomy for LVO stroke, as younger age was significantly associated with increased brain edema formation.^60^

Our meta‐analysis had several limitations. First, there have been no randomized controlled trials comparing the safety and efficacy of aspiration and stent retriever in treating posterior circulation stroke. All the studies included in this meta‐analysis were cohort studies. Nine studies^24^, ^25^, ^26^, ^27^, ^28^, ^30^, ^32^, ^34^, ^37^ were conducted retrospectively; therefore, any conclusions drawn were subject to the limitations of the retrospective study design, including recall and observer bias. Furthermore, the length of follow‐up varied between the nine studies; subacute and late complications were more likely to be reported in studies with longer follow‐up periods. Second, some critical confounders, such as baseline NIHSS score, devices used, vascular occlusion sites, causative mechanisms of stroke, pre‐stroke imaging selection, and selection criteria for thrombectomy were not balanced between the aspiration and stent retriever groups, which may cause a bias in our results regarding favorable outcomes, recanalization rate, duration of the procedure, and complications. Third, according to the previous study, the causative mechanism is a very important confounder in the treatment of posterior circulation stroke.^21^ However, due to the lack of patient‐level data, further analysis could not be performed to identify the safety and efficacy of aspiration and stent retrievers based on different causative mechanisms, such as ICAD and non‐ICAD basilar artery occlusion. Therefore, evidence from studies based on different etiologies is required to further assess the differences in efficacy and safety between these techniques in patients with posterior circulation stroke.

In this meta‐analysis, mechanical thrombectomy using aspiration was associated with a better recanalization rate, favorable outcome, first‐pass effect, and shorter procedure duration than those of stent retriever in posterior circulation stroke. However, due to the limitations of the study, the conclusion should be interpreted cautiously. Future randomized controlled trials with larger sample sizes are warranted to confirm this finding.

## CONFLICT OF INTEREST

The authors have declared that no competing interest exists.

## Supporting information


AppendixS1

## Data Availability

Data sharing not applicable ‐ no new data generated.
