# Supplementary material for: Aspiration versus stent retriever for posterior circulation stroke: A meta‐analysis
Source: CNS Neurosci Ther. 2022 Dec 13;29(2):525–37. doi: 10.1111/cns.14045 (PMC9873527; doi:10.1111/cns.14045)
Supplement: Supplementary file 1 — AppendixS1 [file CNS-29-525-s001.pdf]

SUPPLEMENTAL MATERIAL

Aspiration Versus Stent Retriever for Posterior Circulation Stroke: A Meta-Analysis

Detailed search query

Supplement Figure 1 Forest plot for successful recanalization, complete recanalization and first pass effect in basilar artery occlusion.

Supplement Figure 2 Forest plot for total complication in basilar artery occlusion, subgroup for various complications and subgroup for different hemorrhagic types. sICH: symptomatic intracranial hemorrhage.

Supplement Figure 3 Forest plot for secondary outcomes in basilar artery occlusion.

Supplement Figure 4 Forest plot for outcomes based on East-Asian group and Non-East Asian group.

Detailed search query

1. **PubMed**

(Final search run on 16-04-2022; 59 items)

((stent*[Title/Abstract]) AND ((((((((((aspiration*[Title/Abstract]) OR (Percutaneous Aspiration Thrombectomy[Title/Abstract])) OR (Aspiration Thrombectomies, Percutaneous[Title/Abstract])) OR (Aspiration Thrombectomy, Percutaneous[Title/Abstract])) OR (Percutaneous Aspiration Thrombectomies[Title/Abstract])) OR (Thrombectomies, Percutaneous Aspiration[Title/Abstract])) OR (Thrombectomy, Percutaneous Aspiration[Title/Abstract])) OR (Aspiration Thrombectomy[Title/Abstract])) OR (Aspiration Thrombectomies[Title/Abstract])) OR (Thrombectomies, Aspiration[Title/Abstract])) OR (Thrombectomy, Aspiration[Title/Abstract]))) AND (((((((((Posterior Circulation Stroke[MeSH Terms]) OR (Posterior Circulation Stroke[Title/Abstract])) OR (Brain Infarction, Posterior Circulation[Title/Abstract])) OR (Posterior Circulation Infarction, Brain[Title/Abstract])) OR (Posterior Circulation Brain Infarction[Title/Abstract])) OR (Infarction, Brain, Posterior Circulation[Title/Abstract])) OR (Infarction, Posterior Circulation, Brain[Title/Abstract])) OR (Vertebral artery occlusion[Title/Abstract])) OR (posterior cerebral artery occlusion[Title/Abstract])) OR (basilar artery occlusion[Title/Abstract]))

2. **Embase**

(Final search run on 16-04-2022; 60 items)

((‘stent*’.ti,ab) AND ((((((((((‘aspiration*’.ti,ab.) OR (‘Percutaneous Aspiration Thrombectomy’.ti,ab.)) OR (‘Aspiration Thrombectomies, Percutaneous’.ti,ab.)) OR (‘Aspiration Thrombectomy, Percutaneous’.ti,ab.)) OR (‘Percutaneous Aspiration Thrombectomies’.ti,ab.)) OR (‘Thrombectomies, Percutaneous Aspiration’.ti,ab.)) OR (‘Thrombectomy, Percutaneous Aspiration’.ti,ab.)) OR (‘Aspiration Thrombectomy’.ti,ab.)) OR (‘Aspiration Thrombectomies’.ti,ab.)) OR (‘Thrombectomies, Aspiration’.ti,ab.)) OR (‘Thrombectomy, Aspiration’.ti,ab.))) AND (((((((((exp*’Posterior Circulation Stroke’) OR (Posterior Circulation Stroke’.ti,ab.)) OR (Brain Infarction, Posterior Circulation’.ti,ab.)) OR (‘Posterior Circulation Infarction, Brain’.ti,ab.)) OR (‘Posterior Circulation Brain Infarction’.ti,ab.)) OR (‘Infarction, Brain, Posterior Circulation’.ti,ab.)) OR (‘Infarction, Posterior Circulation, Brain’.ti,ab.)) OR (‘Vertebral artery occlusion’.ti,ab.)) OR (‘posterior cerebral artery occlusion’.ti,ab.)) OR (‘basilar artery occlusion’.ti,ab.))

3. **Cochrane**

(Final search run on 16-04-2022; 15 items)

((stent*[Title/Abstract]) AND ((((((((((aspiration*[Title/Abstract]) OR (Percutaneous Aspiration Thrombectomy[Title/Abstract])) OR (Aspiration Thrombectomies, Percutaneous[Title/Abstract])) OR (Aspiration Thrombectomy, Percutaneous[Title/Abstract])) OR (Percutaneous Aspiration Thrombectomies[Title/Abstract])) OR (Thrombectomies, Percutaneous Aspiration[Title/Abstract])) OR (Thrombectomy, Percutaneous Aspiration[Title/Abstract])) OR (Aspiration Thrombectomy[Title/Abstract])) OR (Aspiration Thrombectomies[Title/Abstract])) OR (Thrombectomies, Aspiration[Title/Abstract])) OR (Thrombectomy, Aspiration[Title/Abstract]))) AND ((((((((((Posterior Circulation Stroke[MeSH Terms]) OR (Posterior Circulation Stroke[Title/Abstract])) OR (Brain Infarction, Posterior Circulation[Title/Abstract])) OR (Posterior Circulation Infarction, Brain[Title/Abstract])) OR (Posterior Circulation Brain Infarction[Title/Abstract])) OR (Infarction, Brain, Posterior Circulation[Title/Abstract])) OR (Infarction, Posterior Circulation, Brain[Title/Abstract])) OR (Vertebral artery occlusion[Title/Abstract])) OR (posterior cerebral artery occlusion[Title/Abstract])) OR (basilar artery occlusion[Title/Abstract]))

A. Successful recanalization

Study or Subgroup	aspiration Events	aspiration Total	stent retrieve Events	stent retrieve Total	Weight	Odds Ratio M-H, Random, 95% CI
Abdelrady 2022	27	33	27	35	14.3%	1.33 [0.41, 4.36]
Baik 2021	37	43	97	118	20.8%	1.34 [0.50, 3.57]
Choi 2020	14	16	25	34	7.3%	2.52 [0.48, 13.33]
Giorgianni 2018	20	27	45	60	18.6%	0.95 [0.34, 2.70]
Gory 2018	40	46	39	54	18.4%	2.56 [0.90, 7.29]
Kang 2018	63	67	131	145	15.2%	1.68 [0.53, 5.32]
Lee 2018	3	5	4	6	3.3%	0.75 [0.06, 8.83]
Son 2016	18	18	11	13	2.1%	8.04 [0.35, 182.94]
**Total (95% CI)**		**255**		**465**	**100.0%**	**1.56 [1.00, 2.45]**
Total events	222		379			

Heterogeneity: $Tau^2 = 0.00$; $Chi^2 = 3.64$, df = 7 (P = 0.82); $I^2 = 0\%$
Test for overall effect: Z = 1.95 (P = 0.05)

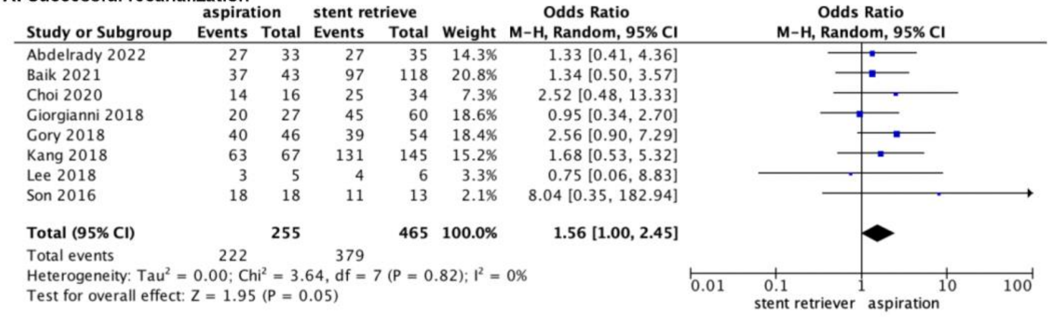

B. Complete recanalization

Study or Subgroup	aspiration Events	aspiration Total	stent retriever Events	stent retriever Total	Weight	Odds Ratio M-H, Random, 95% CI
Baik 2021	30	43	56	118	26.3%	2.55 [1.21, 5.38]
Gory 2018	25	46	17	54	24.8%	2.59 [1.15, 5.86]
Kang 2018	41	67	93	145	29.2%	0.88 [0.49, 1.60]
Lee 2018	3	5	3	6	7.2%	1.50 [0.14, 16.54]
Son 2016	13	18	3	13	12.5%	8.67 [1.66, 45.21]
**Total (95% CI)**		**179**		**336**	**100.0%**	**2.11 [1.03, 4.33]**
Total events	112		172			

Heterogeneity: $Tau^2 = 0.37$; $Chi^2 = 10.56$, df = 4 (P = 0.03); $I^2 = 62\%$
Test for overall effect: Z = 2.03 (P = 0.04)

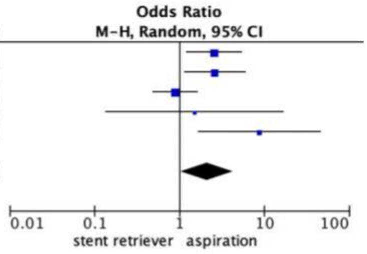

C. First pass effect

Study or Subgroup	aspiration Events	aspiration Total	stent retriever Events	stent retriever Total	Weight	Odds Ratio M-H, Random, 95% CI
Abdelrady 2022	15	33	10	35	29.7%	2.08 [0.76, 5.68]
Baik 2021	17	43	23	118	51.5%	2.70 [1.26, 5.79]
Choi 2020	11	16	13	34	18.8%	3.55 [1.00, 12.57]
**Total (95% CI)**		**92**		**187**	**100.0%**	**2.63 [1.52, 4.55]**
Total events	43		46			

Heterogeneity: $Tau^2 = 0.00$; $Chi^2 = 0.43$, df = 2 (P = 0.81); $I^2 = 0\%$
Test for overall effect: Z = 3.47 (P = 0.0005)

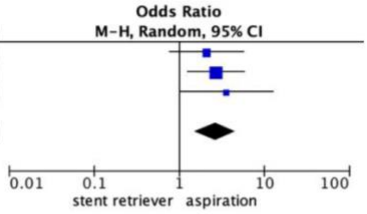

Supplement Figure 1 Forest plot for successful recanalization, complete recanalization and first pass effect.

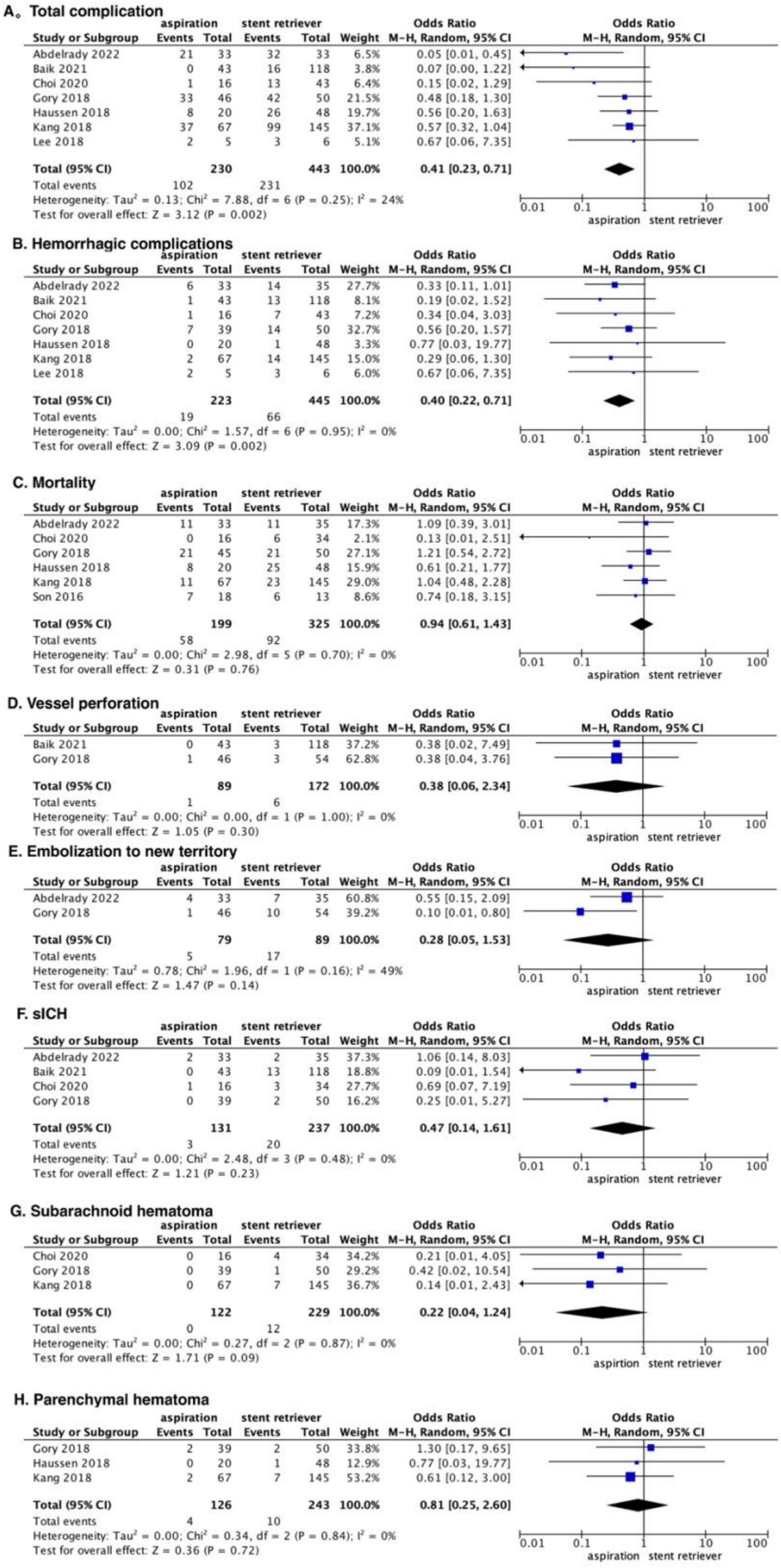

Supplement Figure 2 Forest plot for total complication in basilar artery occlusion, subgroup for various complications and subgroup for different hemorrhagic types. sICH: symptomatic intracranial hemorrhage

A. Favorable outcome

Study or Subgroup	aspiration Events	aspiration Total	stent retrive Events	stent retrive Total	Weight	Odds Ratio M-H, Random, 95% CI
Abdelrady 2022	14	33	12	35	9.4%	1.41 [0.53, 3.77]
Baik 2021	17	43	38	118	17.4%	1.38 [0.67, 2.84]
Choi 2020	9	16	12	34	6.2%	2.36 [0.70, 7.93]
Kaneko 2021	8	21	15	38	7.6%	0.94 [0.32, 2.82]
Giorgianni 2018	3	27	4	60	3.7%	1.75 [0.36, 8.42]
Gory 2018	18	45	18	50	13.2%	1.19 [0.52, 2.72]
Haussen 2018	11	20	15	48	7.9%	2.69 [0.92, 7.85]
Kang 2018	27	67	68	145	26.3%	0.76 [0.42, 1.37]
Lee 2018	1	5	3	5	1.1%	0.17 [0.01, 2.82]
Li 2017	5	7	20	50	3.0%	3.75 [0.66, 21.25]
Son 2016	8	18	5	13	4.3%	1.28 [0.30, 5.48]
**Total (95% CI)**		**302**		**596**	**100.0%**	**1.24 [0.92, 1.68]**
Total events	121		210			

Heterogeneity: $Tau^2 = 0.00$; $Chi^2 = 9.77$, df = 10 (P = 0.46); $I^2 = 0\%$
Test for overall effect: Z = 1.41 (P = 0.16)

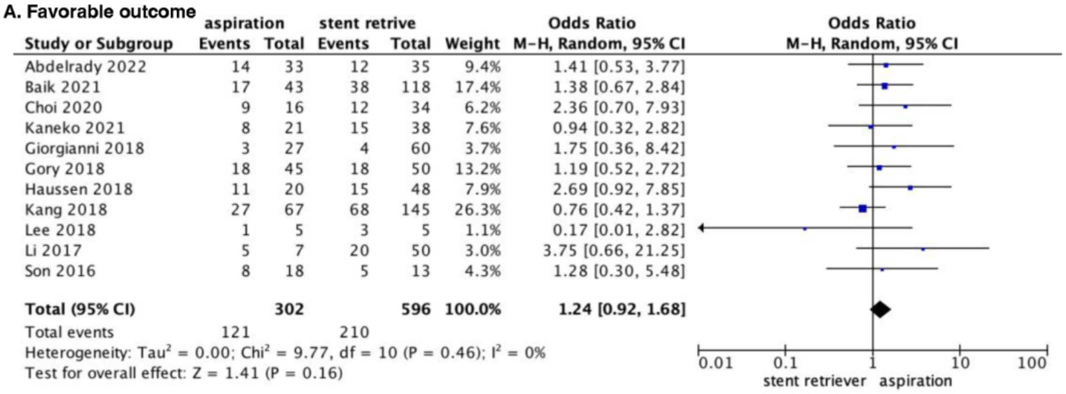

B. Rescue therapy

Study or Subgroup	aspiraton Events	aspiraton Total	stent retriever Events	stent retriever Total	Weight	Odds Ratio M-H, Random, 95% CI
Abdelrady 2022	20	53	10	39	36.0%	1.76 [0.71, 4.36]
Gory 2018	12	46	2	54	23.5%	9.18 [1.93, 43.59]
Kang 2018	15	67	32	145	40.5%	1.02 [0.51, 2.04]
**Total (95% CI)**		**166**		**238**	**100.0%**	**2.08 [0.73, 5.89]**
Total events	47		44			

Heterogeneity: $Tau^2 = 0.57$; $Chi^2 = 6.58$, df = 2 (P = 0.04); $I^2 = 70\%$
Test for overall effect: Z = 1.38 (P = 0.17)

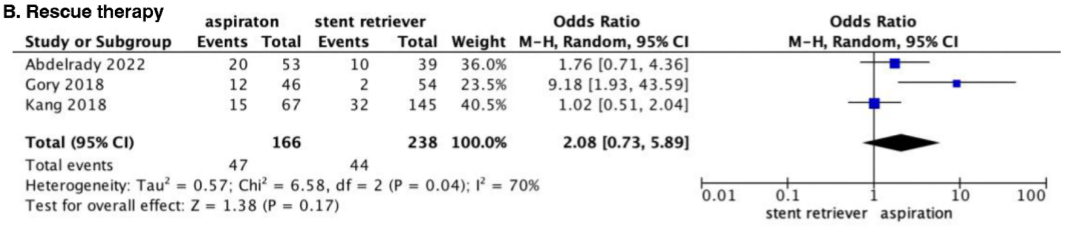

Supplement Figure 3 Forest plot for secondary outcomes in basilar artery occlusion.

A. Successul recanalization

Study or Subgroup	aspiration Events	aspiration Total	stent retriever Events	stent retriever Total	Weight	Odds Ratio M-H, Random, 95% CI
**12.1.1 East-Asian**						
Baik 2021	37	43	97	118	12.3%	1.34 [0.50, 3.57]
Choi 2020	14	16	25	34	4.3%	2.52 [0.48, 13.33]
Haussen 2018	63	67	131	145	9.0%	1.68 [0.53, 5.32]
Kang 2018	3	5	4	7	2.2%	1.13 [0.11, 11.60]
Lee 2018	18	18	11	13	1.2%	8.04 [0.35, 182.94]
**Subtotal (95% CI)**		**149**		**317**	**28.9%**	**1.68 [0.88, 3.18]**
Total events	135		268			
Heterogeneity: $Tau^2 = 0.00$; $Chi^2 = 1.52$, df = 4 (P = 0.82); $I^2 = 0\%$						
Test for overall effect: Z = 1.58 (P = 0.11)						
**12.1.2 Non-East Asian**						
Bernsen 2022	60	71	93	134	21.6%	2.40 [1.15, 5.04]
Abdelrady 2022	27	33	27	35	8.4%	1.33 [0.41, 4.36]
Onodera 2021	22	23	10	11	1.4%	2.20 [0.12, 38.83]
Giorgianni 2018	20	27	45	60	11.0%	0.95 [0.34, 2.70]
Gory 2018	40	56	39	54	17.2%	0.96 [0.42, 2.21]
Mokin 2016	35	42	45	58	11.4%	1.44 [0.52, 4.00]
**Subtotal (95% CI)**		**252**		**352**	**71.1%**	**1.43 [0.95, 2.16]**
Total events	204		259			
Heterogeneity: $Tau^2 = 0.00$; $Chi^2 = 3.46$, df = 5 (P = 0.63); $I^2 = 0\%$						
Test for overall effect: Z = 1.72 (P = 0.08)						
**Total (95% CI)**		**401**		**669**	**100.0%**	**1.50 [1.06, 2.12]**
Total events	339		527			
Heterogeneity: $Tau^2 = 0.00$; $Chi^2 = 5.15$, df = 10 (P = 0.88); $I^2 = 0\%$						
Test for overall effect: Z = 2.30 (P = 0.02)						
Test for subgroup differences: $Chi^2 = 0.17$, df = 1 (P = 0.68), $I^2 = 0\%$						

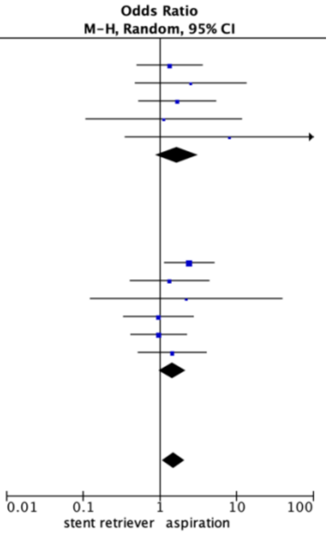

B. First pass effect

Study or Subgroup	aspiration Events	aspiration Total	stent retriever Events	stent retriever Total	Weight	Odds Ratio M-H, Random, 95% CI
**12.2.1 East Asian**						
Baik 2021	17	43	23	118	25.7%	2.70 [1.26, 5.79]
Choi 2020	11	16	13	34	9.4%	3.55 [1.00, 12.57]
**Subtotal (95% CI)**		**59**		**152**	**35.0%**	**2.91 [1.51, 5.58]**
Total events	28		36			
Heterogeneity: $Tau^2 = 0.00$; $Chi^2 = 0.13$, df = 1 (P = 0.72); $I^2 = 0\%$						
Test for overall effect: Z = 3.20 (P = 0.001)						
**12.2.2 Non-East Asian**						
Bernsen 2022	44	71	66	134	43.3%	1.68 [0.93, 3.02]
Abdelrady 2022	15	33	10	35	14.8%	2.08 [0.76, 5.68]
Onodera 2021	13	23	4	11	6.8%	2.27 [0.52, 9.99]
**Subtotal (95% CI)**		**127**		**180**	**65.0%**	**1.82 [1.13, 2.94]**
Total events	72		80			
Heterogeneity: $Tau^2 = 0.00$; $Chi^2 = 0.23$, df = 2 (P = 0.89); $I^2 = 0\%$						
Test for overall effect: Z = 2.45 (P = 0.01)						
**Total (95% CI)**		**186**		**332**	**100.0%**	**2.14 [1.46, 3.16]**
Total events	100		116			
Heterogeneity: $Tau^2 = 0.00$; $Chi^2 = 1.64$, df = 4 (P = 0.80); $I^2 = 0\%$						
Test for overall effect: Z = 3.87 (P = 0.0001)						
Test for subgroup differences: $Chi^2 = 1.28$, df = 1 (P = 0.26), $I^2 = 21.9\%$						

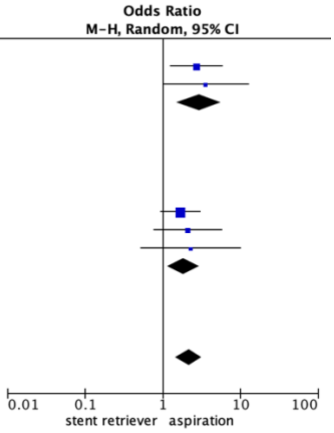

C. Complete recanalization

Study or Subgroup	aspiration Events	aspiration Total	stent retriever Events	stent retriever Total	Weight	Odds Ratio M-H, Random, 95% CI
**12.3.1 East-Asian**						
Baik 2021	30	43	56	118	17.9%	2.55 [1.21, 5.38]
Kang 2018	41	67	93	145	23.1%	0.88 [0.49, 1.60]
Lee 2018	3	5	3	7	2.7%	2.00 [0.19, 20.61]
**Subtotal (95% CI)**		**115**		**270**	**43.7%**	**1.50 [0.64, 3.52]**
Total events	74		152			
Heterogeneity: $Tau^2 = 0.30$; $Chi^2 = 4.89$, df = 2 (P = 0.09); $I^2 = 59\%$						
Test for overall effect: Z = 0.94 (P = 0.35)						
**12.3.2 Non-East Asian**						
Bernsen 2022	36	71	50	134	23.7%	1.73 [0.97, 3.09]
Gory 2018	70	127	52	107	26.7%	1.30 [0.78, 2.18]
Onodera 2021	17	23	4	11	5.8%	4.96 [1.06, 23.16]
**Subtotal (95% CI)**		**221**		**252**	**56.3%**	**1.65 [1.03, 2.65]**
Total events	123		106			
Heterogeneity: $Tau^2 = 0.05$; $Chi^2 = 2.76$, df = 2 (P = 0.25); $I^2 = 28\%$						
Test for overall effect: Z = 2.06 (P = 0.04)						
**Total (95% CI)**		**336**		**522**	**100.0%**	**1.57 [1.06, 2.33]**
Total events	197		258			
Heterogeneity: $Tau^2 = 0.08$; $Chi^2 = 7.90$, df = 5 (P = 0.16); $I^2 = 37\%$						
Test for overall effect: Z = 2.23 (P = 0.03)						
Test for subgroup differences: $Chi^2 = 0.03$, df = 1 (P = 0.85), $I^2 = 0\%$						

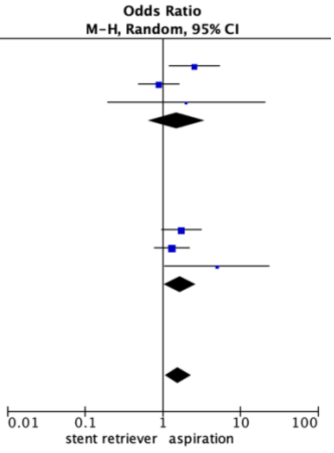

D. Total complication

Study or Subgroup	aspiration Events	aspiration Total	stent retriever Events	stent retriever Total	Weight	Odds Ratio M-H, Random, 95% CI
**12.4.1 East-Asian**						
Baik 2021	0	43	16	118	2.6%	0.07 [0.00, 1.22]
Choi 2020	1	16	13	43	4.4%	0.15 [0.02, 1.29]
Haussen 2018	8	20	26	48	14.3%	0.56 [0.20, 1.63]
Kang 2018	37	67	99	145	29.0%	0.57 [0.32, 1.04]
Lee 2018	2	5	4	7	3.7%	0.50 [0.05, 5.15]
**Subtotal (95% CI)**		**151**		**361**	**54.0%**	**0.50 [0.31, 0.81]**
Total events	48		158			
Heterogeneity: $Tau^2 = 0.00$; $Chi^2 = 3.52$, df = 4 (P = 0.47); $I^2 = 0\%$						
Test for overall effect: Z = 2.81 (P = 0.005)						
**12.4.2 Non-East Asian**						
Bernsen 2022	68	71	133	134	3.9%	0.17 [0.02, 1.67]
Abdelrady 2022	21	33	32	33	4.5%	0.05 [0.01, 0.45]
Onodera 2021	12	23	6	11	8.8%	0.91 [0.22, 3.84]
De La Riva 2018	7	21	13	40	13.1%	1.04 [0.34, 3.19]
Gory 2018	33	46	42	50	15.8%	0.48 [0.18, 1.30]
**Subtotal (95% CI)**		**194**		**268**	**46.0%**	**0.45 [0.18, 1.11]**
Total events	141		226			
Heterogeneity: $Tau^2 = 0.47$; $Chi^2 = 7.58$, df = 4 (P = 0.11); $I^2 = 47\%$						
Test for overall effect: Z = 1.74 (P = 0.08)						
**Total (95% CI)**		**345**		**629**	**100.0%**	**0.48 [0.30, 0.76]**
Total events	189		384			
Heterogeneity: $Tau^2 = 0.10$; $Chi^2 = 11.12$, df = 9 (P = 0.27); $I^2 = 19\%$						
Test for overall effect: Z = 3.10 (P = 0.002)						
Test for subgroup differences: $Chi^2 = 0.04$, df = 1 (P = 0.85), $I^2 = 0\%$						

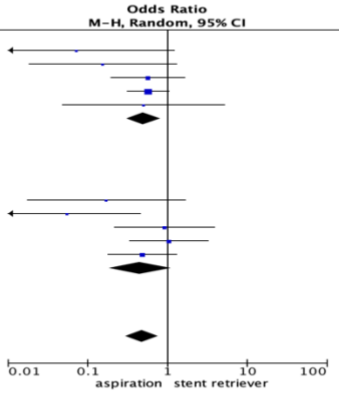

E. Hemorrhagic complication

Study or Subgroup	aspiration Events	aspiration Total	stent retriever Events	stent retriever Total	Weight	Odds Ratio M-H, Random, 95% CI
**12.5.1 East-Asian**						
Baik 2021	0	43	13	118	3.0%	0.09 [0.01, 1.54]
Choi 2020	1	16	7	43	5.2%	0.34 [0.04, 3.03]
Haussen 2018	0	20	1	48	2.3%	0.77 [0.03, 19.77]
Kang 2018	2	67	14	145	10.8%	0.29 [0.06, 1.30]
Lee 2018	2	5	4	7	4.5%	0.50 [0.05, 5.15]
**Subtotal (95% CI)**		**151**		**361**	**25.8%**	**0.31 [0.12, 0.83]**
Total events	5		39			
Heterogeneity: Tau² = 0.00; Chi² = 1.30, df = 4 (P = 0.86); I² = 0%						
Test for overall effect: Z = 2.33 (P = 0.02)						
**12.5.2 Non-East Asian**						
Bernsen 2022	2	71	5	135	8.8%	0.75 [0.14, 3.99]
Abdelrady 2022	6	33	14	35	19.8%	0.33 [0.11, 1.01]
Onodera 2021	11	23	5	11	11.8%	1.10 [0.26, 4.65]
De La Riva 2018	3	21	5	40	10.4%	1.17 [0.25, 5.44]
Gory 2018	7	39	14	50	23.4%	0.56 [0.20, 1.57]
**Subtotal (95% CI)**		**187**		**271**	**74.2%**	**0.62 [0.35, 1.11]**
Total events	29		43			
Heterogeneity: Tau² = 0.00; Chi² = 2.54, df = 4 (P = 0.64); I² = 0%						
Test for overall effect: Z = 1.61 (P = 0.11)						
**Total (95% CI)**		**338**		**632**	**100.0%**	**0.52 [0.32, 0.86]**
Total events	34		82			
Heterogeneity: Tau² = 0.00; Chi² = 5.32, df = 9 (P = 0.81); I² = 0%						
Test for overall effect: Z = 2.57 (P = 0.01)						
Test for subgroup differences: Chi² = 1.42, df = 1 (P = 0.23), I² = 29.8%						

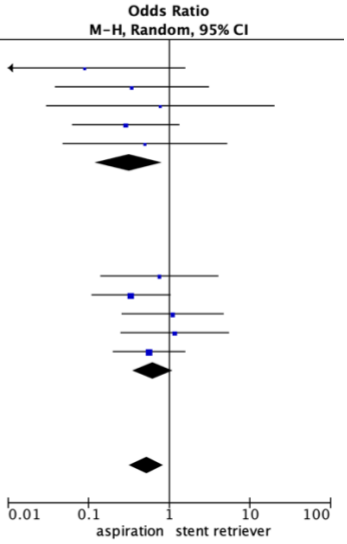

F. Mortality

Study or Subgroup	aspiration Events	aspiration Total	stent retriever Events	stent retriever Total	Weight	Odds Ratio M-H, Random, 95% CI
**12.6.1 East-Asian**						
Choi 2020	0	16	6	34	1.3%	0.13 [0.01, 2.51]
Haussen 2018	8	20	25	48	9.8%	0.61 [0.21, 1.77]
Kang 2018	11	67	23	145	17.8%	1.04 [0.48, 2.28]
Son 2016	7	18	6	13	5.3%	0.74 [0.18, 3.15]
**Subtotal (95% CI)**		**121**		**240**	**34.2%**	**0.79 [0.45, 1.39]**
Total events	26		60			
Heterogeneity: Tau² = 0.00; Chi² = 2.16, df = 3 (P = 0.54); I² = 0%						
Test for overall effect: Z = 0.83 (P = 0.41)						
**12.6.2 Non-East Asian**						
Bernsen 2022	31	71	55	134	32.5%	1.11 [0.62, 1.99]
Abdelrady 2022	11	33	11	35	10.6%	1.09 [0.39, 3.01]
De La Riva 2018	4	21	7	40	5.9%	1.11 [0.28, 4.32]
Gory 2018	21	45	21	50	16.7%	1.21 [0.54, 2.72]
**Subtotal (95% CI)**		**170**		**259**	**65.8%**	**1.13 [0.75, 1.70]**
Total events	67		94			
Heterogeneity: Tau² = 0.00; Chi² = 0.03, df = 3 (P = 1.00); I² = 0%						
Test for overall effect: Z = 0.60 (P = 0.55)						
**Total (95% CI)**		**291**		**499**	**100.0%**	**1.00 [0.72, 1.39]**
Total events	93		154			
Heterogeneity: Tau² = 0.00; Chi² = 3.22, df = 7 (P = 0.86); I² = 0%						
Test for overall effect: Z = 0.00 (P = 1.00)						
Test for subgroup differences: Chi² = 1.04, df = 1 (P = 0.31), I² = 4.1%						

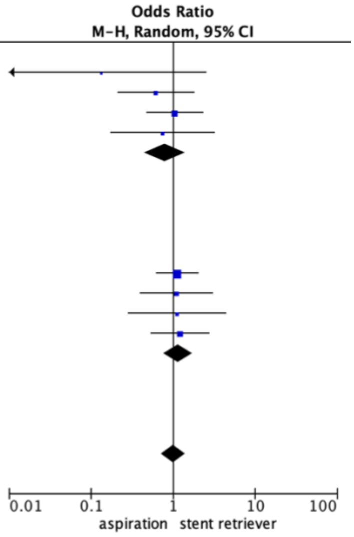

G. Vessel perforration

Study or Subgroup	aspiration Events	aspiration Total	stent retriever Events	stent retriever Total	Weight	Odds Ratio M-H, Random, 95% CI
**12.7.1 East-Asian**						
Baik 2021	0	43	3	118	37.2%	0.38 [0.02, 7.49]
**Subtotal (95% CI)**		**43**		**118**	**37.2%**	**0.38 [0.02, 7.49]**
Total events	0		3			
Heterogeneity: Not applicable						
Test for overall effect: Z = 0.64 (P = 0.52)						
**12.7.2 Non-East Asian**						
Gory 2018	1	46	3	54	62.8%	0.38 [0.04, 3.76]
**Subtotal (95% CI)**		**46**		**54**	**62.8%**	**0.38 [0.04, 3.76]**
Total events	1		3			
Heterogeneity: Not applicable						
Test for overall effect: Z = 0.83 (P = 0.41)						
**Total (95% CI)**		**89**		**172**	**100.0%**	**0.38 [0.06, 2.34]**
Total events	1		6			
Heterogeneity: Tau² = 0.00; Chi² = 0.00, df = 1 (P = 1.00); I² = 0%						
Test for overall effect: Z = 1.05 (P = 0.30)						
Test for subgroup differences: Chi² = 0.00, df = 1 (P = 1.00), I² = 0%						

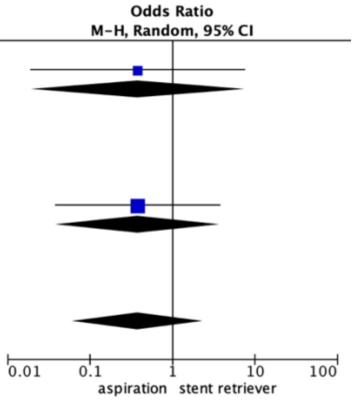

H. sICH

Study or Subgroup	aspiration Events	aspiration Total	stent retriever Events	stent retriever Total	Weight	Odds Ratio M-H, Random, 95% CI
**12.9.1 East Asian**						
Baik 2021	0	43	13	118	10.5%	0.09 [0.01, 1.54]
Choi 2020	1	16	3	34	15.5%	0.69 [0.07, 7.19]
**Subtotal (95% CI)**		**59**		**152**	**26.0%**	**0.29 [0.04, 2.36]**
Total events	1		16			
Heterogeneity: Tau² = 0.58; Chi² = 1.33, df = 1 (P = 0.25); I² = 25%						
Test for overall effect: Z = 1.16 (P = 0.25)						
**12.9.2 Non-East Asian**						
Bernsen 2022	2	71	5	134	30.7%	0.75 [0.14, 3.96]
Abdelrady 2022	2	33	2	35	20.8%	1.06 [0.14, 8.03]
Onodera 2021	2	23	1	11	13.4%	0.95 [0.08, 11.79]
Gory 2018	0	39	2	50	9.1%	0.25 [0.01, 5.27]
**Subtotal (95% CI)**		**166**		**230**	**74.0%**	**0.75 [0.26, 2.20]**
Total events	6		10			
Heterogeneity: Tau² = 0.00; Chi² = 0.67, df = 3 (P = 0.88); I² = 0%						
Test for overall effect: Z = 0.52 (P = 0.60)						
**Total (95% CI)**		**225**		**382**	**100.0%**	**0.59 [0.24, 1.49]**
Total events	7		26			
Heterogeneity: Tau² = 0.00; Chi² = 2.86, df = 5 (P = 0.72); I² = 0%						
Test for overall effect: Z = 1.11 (P = 0.27)						
Test for subgroup differences: Chi² = 0.64, df = 1 (P = 0.42), I² = 0%						

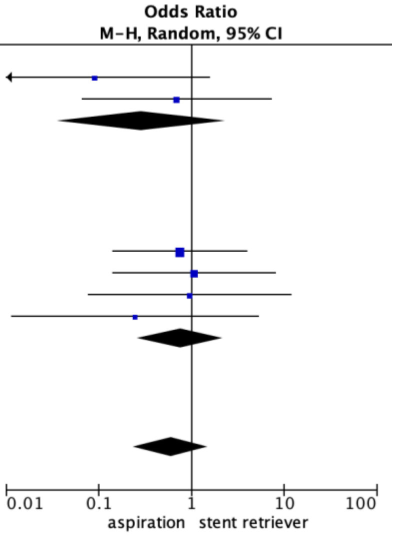

I. Subarachnoid hematoma

Study or Subgroup	aspiration Events	aspiration Total	stent retriever Events	stent retriever Total	Weight	Odds Ratio M-H, Random, 95% CI
**12.10.1 East-Asian**						
Haussen 2018	0	20	1	48	12.9%	0.77 [0.03, 19.77]
Kang 2018	2	67	7	145	53.2%	0.61 [0.12, 3.00]
**Subtotal (95% CI)**		**87**		**193**	**66.2%**	**0.64 [0.15, 2.67]**
Total events	2		8			
Heterogeneity: Tau² = 0.00; Chi² = 0.02, df = 1 (P = 0.90); I² = 0%						
Test for overall effect: Z = 0.62 (P = 0.54)						
**12.10.2 Non-East Asian**						
Gory 2018	2	39	2	50	33.8%	1.30 [0.17, 9.65]
**Subtotal (95% CI)**		**39**		**50**	**33.8%**	**1.30 [0.17, 9.65]**
Total events	2		2			
Heterogeneity: Not applicable						
Test for overall effect: Z = 0.25 (P = 0.80)						
**Total (95% CI)**		**126**		**243**	**100.0%**	**0.81 [0.25, 2.60]**
Total events	4		10			
Heterogeneity: Tau² = 0.00; Chi² = 0.34, df = 2 (P = 0.84); I² = 0%						
Test for overall effect: Z = 0.36 (P = 0.72)						
Test for subgroup differences: Chi² = 0.32, df = 1 (P = 0.57), I² = 0%						

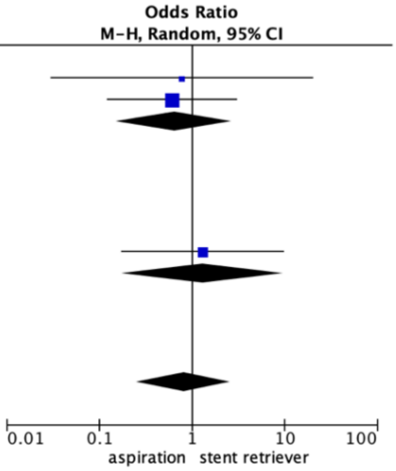

J. Parenchymal hematoma

Study or Subgroup	aspiration Events	aspiration Total	stent retriever Events	stent retriever Total	Weight	Odds Ratio M-H, Random, 95% CI
**12.11.1 East-Asian**						
Choi 2020	0	16	4	34	34.2%	0.21 [0.01, 4.05]
Kang 2018	0	67	7	145	36.7%	0.14 [0.01, 2.43]
**Subtotal (95% CI)**		**83**		**179**	**70.8%**	**0.17 [0.02, 1.32]**
Total events	0		11			
Heterogeneity: Tau² = 0.00; Chi² = 0.04, df = 1 (P = 0.85); I² = 0%						
Test for overall effect: Z = 1.70 (P = 0.09)						
**12.11.2 Non-East Asian**						
Gory 2018	0	39	1	50	29.2%	0.42 [0.02, 10.54]
**Subtotal (95% CI)**		**39**		**50**	**29.2%**	**0.42 [0.02, 10.54]**
Total events	0		1			
Heterogeneity: Not applicable						
Test for overall effect: Z = 0.53 (P = 0.60)						
**Total (95% CI)**		**122**		**229**	**100.0%**	**0.22 [0.04, 1.24]**
Total events	0		12			
Heterogeneity: Tau² = 0.00; Chi² = 0.27, df = 2 (P = 0.87); I² = 0%						
Test for overall effect: Z = 1.71 (P = 0.09)						
Test for subgroup differences: Chi² = 0.22, df = 1 (P = 0.64), I² = 0%						

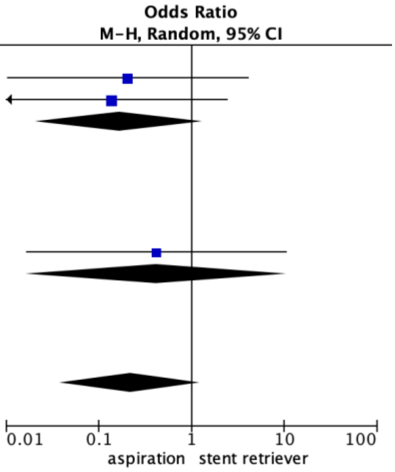

K. Favorable outcome

Study or Subgroup	aspiration Events	aspiration Total	stent retriever Events	stent retriever Total	Weight	Odds Ratio M-H, Random, 95% CI
**12.12.1 East-Asian**						
Baik 2021	17	43	38	118	11.6%	1.38 [0.67, 2.84]
Choi 2020	9	16	12	34	4.1%	2.36 [0.70, 7.93]
Haussen 2018	11	20	15	48	5.3%	2.69 [0.92, 7.85]
Kang 2018	27	67	68	145	17.5%	0.76 [0.42, 1.37]
Lee 2018	1	5	3	6	0.8%	0.25 [0.02, 3.77]
Son 2016	8	18	5	13	2.9%	1.28 [0.30, 5.48]
**Subtotal (95% CI)**		**169**		**364**	**42.2%**	**1.28 [0.78, 2.09]**
Total events	73		141			
Heterogeneity: $Tau^2 = 0.11$; $Chi^2 = 7.06$, df = 5 (P = 0.22); $I^2 = 29\%$						
Test for overall effect: Z = 0.97 (P = 0.33)						
**12.12.2 Non-East Asian**						
Bernsen 2022	30	71	39	134	16.8%	1.78 [0.98, 3.25]
Abdelrady 2022	14	33	12	35	6.3%	1.41 [0.53, 3.77]
Onodera 2021	12	23	3	11	2.5%	2.91 [0.61, 13.83]
Kaneko 2021	8	21	15	38	5.0%	0.94 [0.32, 2.82]
De La Riva 2018	11	21	20	40	5.4%	1.10 [0.38, 3.17]
Giorgianni 2018	3	27	4	60	2.4%	1.75 [0.36, 8.42]
Gory 2018	18	45	18	50	8.8%	1.19 [0.52, 2.72]
Li 2017	5	7	20	50	2.0%	3.75 [0.66, 21.25]
Mokin 2016	14	42	21	58	8.7%	0.88 [0.38, 2.03]
**Subtotal (95% CI)**		**290**		**476**	**57.8%**	**1.39 [1.01, 1.92]**
Total events	115		152			
Heterogeneity: $Tau^2 = 0.00$; $Chi^2 = 4.82$, df = 8 (P = 0.78); $I^2 = 0\%$						
Test for overall effect: Z = 2.01 (P = 0.04)						
**Total (95% CI)**		**459**		**840**	**100.0%**	**1.30 [1.02, 1.67]**
Total events	188		293			
Heterogeneity: $Tau^2 = 0.00$; $Chi^2 = 12.27$, df = 14 (P = 0.58); $I^2 = 0\%$						
Test for overall effect: Z = 2.11 (P = 0.04)						
Test for subgroup differences: $Chi^2 = 0.08$, df = 1 (P = 0.77), $I^2 = 0\%$						

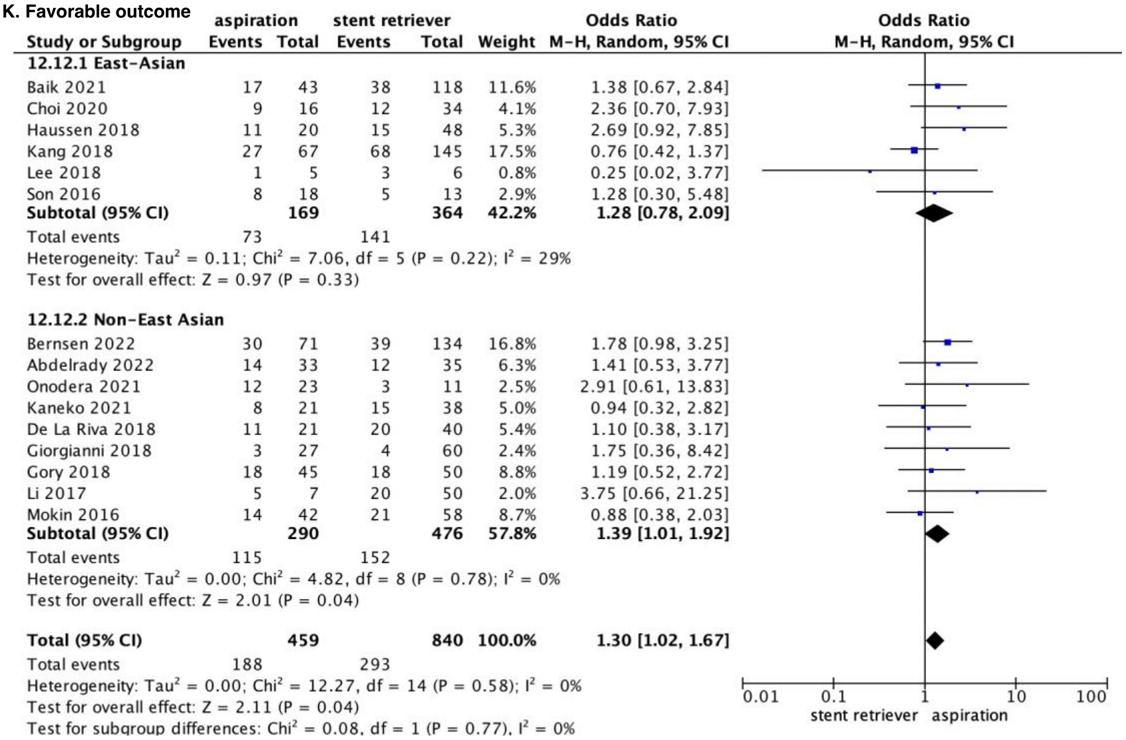

L. Rescue therapy

Study or Subgroup	aspiration Events	aspiration Total	stent retriever Events	stent retriever Total	Weight	Odds Ratio M-H, Random, 95% CI
**12.13.1 East-Asian**						
Kang 2018	15	67	32	145	29.2%	1.02 [0.51, 2.04]
**Subtotal (95% CI)**		**67**		**145**	**29.2%**	**1.02 [0.51, 2.04]**
Total events	15		32			
Heterogeneity: Not applicable						
Test for overall effect: Z = 0.05 (P = 0.96)						
**12.13.2 Non-East Asian**						
Abdelrady 2022	20	53	10	39	25.1%	1.76 [0.71, 4.36]
Onodera 2021	3	23	1	11	8.3%	1.50 [0.14, 16.32]
Gory 2018	12	46	2	54	15.1%	9.18 [1.93, 43.59]
Mokin 2016	6	42	13	58	22.3%	0.58 [0.20, 1.67]
**Subtotal (95% CI)**		**164**		**162**	**70.8%**	**1.80 [0.58, 5.55]**
Total events	41		26			
Heterogeneity: $Tau^2 = 0.81$; $Chi^2 = 8.48$, df = 3 (P = 0.04); $I^2 = 65\%$						
Test for overall effect: Z = 1.02 (P = 0.31)						
**Total (95% CI)**		**231**		**307**	**100.0%**	**1.48 [0.68, 3.22]**
Total events	56		58			
Heterogeneity: $Tau^2 = 0.41$; $Chi^2 = 9.29$, df = 4 (P = 0.05); $I^2 = 57\%$						
Test for overall effect: Z = 0.99 (P = 0.32)						
Test for subgroup differences: $Chi^2 = 0.71$, df = 1 (P = 0.40), $I^2 = 0\%$						

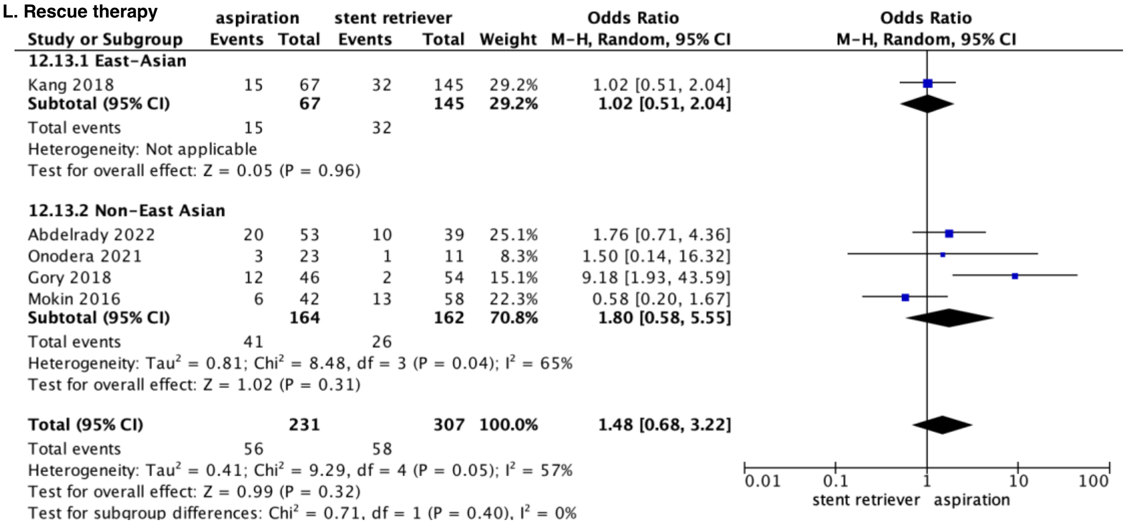

M. Procedure duration

Study or Subgroup	aspiration Mean	aspiration SD	aspiration Total	stent retriever Mean	stent retriever SD	stent retriever Total	Weight	Mean Difference IV, Random, 95% CI
**12.14.1 East-Asian**								
Son 2016	62.3	34.8	18	101.6	41.4	13	32.0%	−39.30 [−66.96, −11.64]
Lee 2018	54.2	41.433	5	82.714	48.207	7	13.7%	−28.51 [−79.45, 22.42]
**Subtotal (95% CI)**			**23**			**20**	**45.7%**	**−36.84 [−61.15, −12.54]**
Heterogeneity: $Tau^2 = 0.00$; $Chi^2 = 0.13$, df = 1 (P = 0.72); $I^2 = 0\%$								
Test for overall effect: Z = 2.97 (P = 0.003)								
**12.14.2 Non-East Asian group**								
Mokin 2016	46	28	42	56	44	58	54.3%	−10.00 [−24.14, 4.14]
**Subtotal (95% CI)**			**42**			**58**	**54.3%**	**−10.00 [−24.14, 4.14]**
Heterogeneity: Not applicable								
Test for overall effect: Z = 1.39 (P = 0.17)								
**Total (95% CI)**			**65**			**78**	**100.0%**	**−21.91 [−42.88, −0.94]**
Heterogeneity: $Tau^2 = 158.72$; $Chi^2 = 3.63$, df = 2 (P = 0.16); $I^2 = 45\%$								
Test for overall effect: Z = 2.05 (P = 0.04)								
Test for subgroup differences: $Chi^2 = 3.50$, df = 1 (P = 0.06), $I^2 = 71.4\%$								

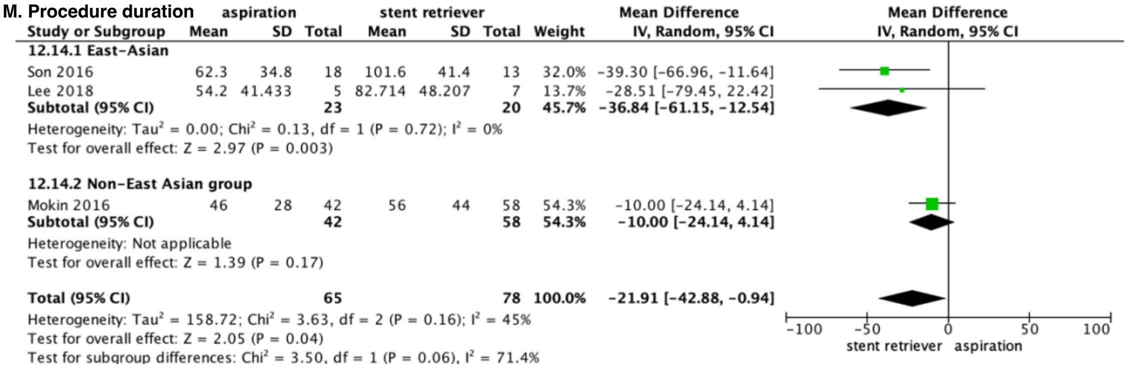

Supplement Figure 4 Forest plot for outcomes based on East-Asian group and Non-East Asian group.
